# Insights into podophyllotoxin lactone features: New cyclolignans as potential dual tubulin‐topoisomerase II inhibitors

**DOI:** 10.1002/ardp.202400600

**Published:** 2024-11-12

**Authors:** Ángela‐Patricia Hernández, Celia Rosales‐Fernández, Carolina Miranda‐Vera, Anzhela Veselinova, Pablo G. Jambrina, Pilar García‐García, Pablo A. García, David Díez, María Ángeles Castro, Manuel Fuentes

**Affiliations:** ^1^ Laboratorio de Química Farmacéutica, Departamento de Ciencias Farmacéuticas, Facultad de Farmacia, CIETUS/IBSAL, Campus Miguel de Unamuno Universidad de Salamanca Salamanca Spain; ^2^ Department of Medicine and General Cytometry Service‐Nucleus, CIBERONC CB16/12/00400, Cancer Research Centre (IBMCC/CSIC/USAL/IBSAL), IBSAL, Campus Miguel de Unamuno Universidad de Salamanca‐CSIC Salamanca Spain; ^3^ Departamento de Química Física, Facultad de Ciencias Químicas Universidad de Salamanca Salamanca Spain; ^4^ Departamento de Química Orgánica, Facultad de Ciencias Químicas Universidad de Salamanca Salamanca Spain; ^5^ Proteomics Unit, Cancer Research Centre (IBMCC/CSIC/USAL/IBSAL) Salamanca Spain

**Keywords:** cytotoxicity, dual‐activity, molecular dynamics, natural products, podophyllic aldehyde

## Abstract

Chemomodulation of natural cyclolignans as podophyllotoxin has been a successful approach to obtain semisynthetic bioactive derivates. One example of this approach is the FDA‐approved drug etoposide for solid and hematological tumors. It differs from the antimitotic activity of the natural product in its mechanism of action, this drug being a topoisomerase II inhibitor instead of a tubulin antimitotic. Within the molecular requirements for the activity of these compounds, the trans‐γ‐lactone moiety presented in the parent compound has always been a feature to be explored to chemomodulate its bioactivity. In this study, we have obtained different compounds that comply with the molecular characteristics for antitubulin and antitopoisomerase II activity combined in a single molecule. Furthermore, we explored the influence of the *trans*‐lactone moiety on the final activity, finding that the *cis*‐lactone was also interesting in terms of bioactivity. The best values of cytotoxicity and cell cycle inhibition were obtained for a compound lacking the lactone ring, thus mimicking the podophyllic aldehyde functionalization, a selective antimitotic podophyllotoxin derivate. The analogs with *cis*‐lactone also presented interesting cytotoxic activity. The present study illustrates the potential of the chemomodulation of natural products such as natural cyclolignan podophyllotoxin derivates for the discovery of new antitumor agents.

## Graphical abstract

Podophyllotoxin is a natural product with interesting antitumor properties that still presents enormous possibilities for chemomodulation of its activity. New strategies of structural modification were explored, targeting both cyclolignan mechanisms of action, tubulin polymerization, and topo II inhibition, finding interesting cytotoxic properties through biological and molecular docking assays.

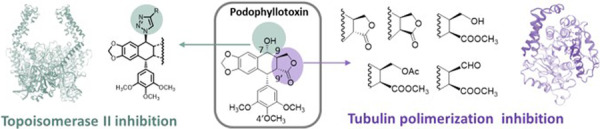

## 1 INTRODUCTION

**Figure 1:**
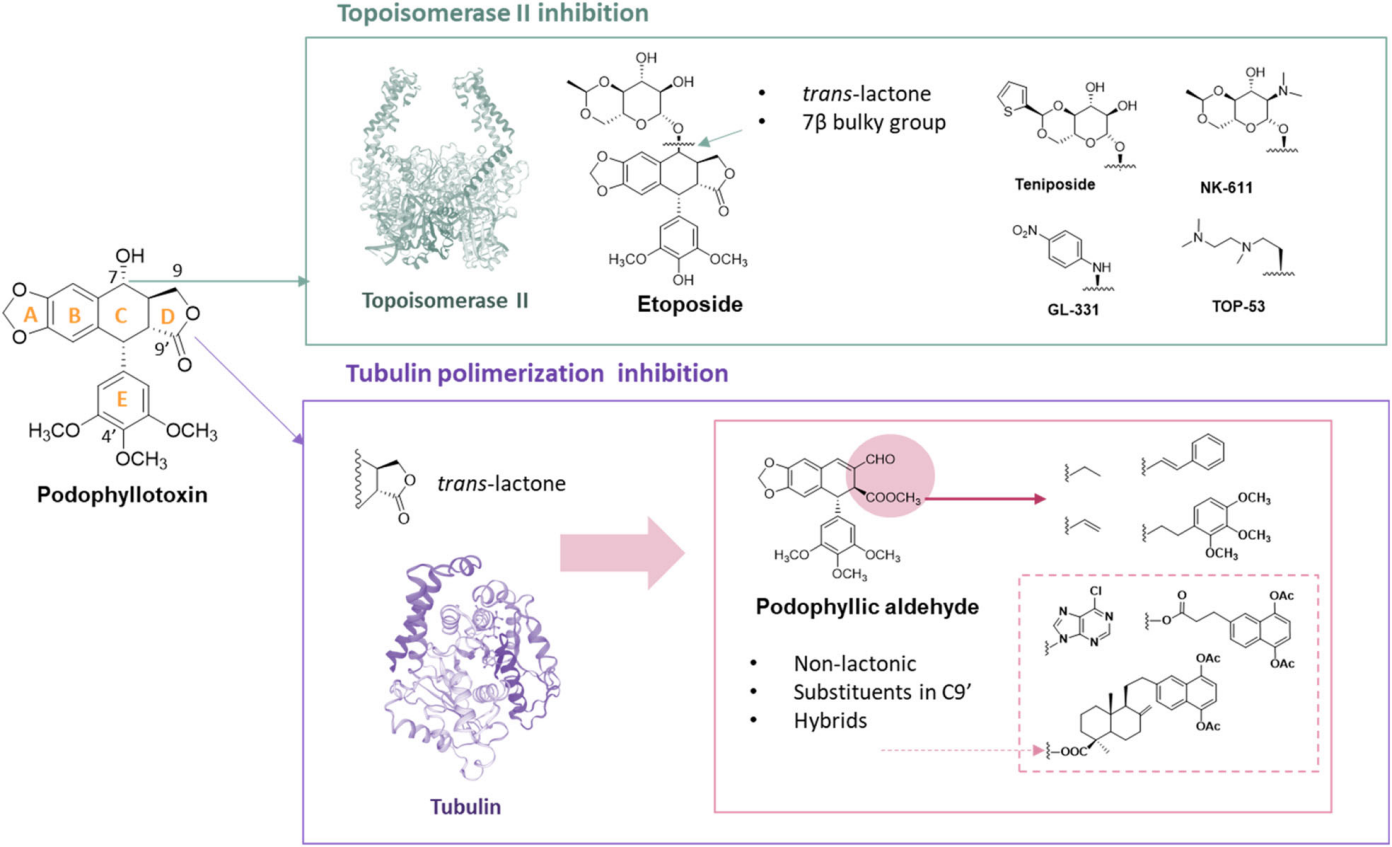
Schematic representation of natural product podophyllotoxin pointing out the molecular features (rings in letters and crucial positions indicated with numbers). This scheme highlights the chemomodulation of podophyllotoxin that has given rise to other active compounds with antitopoisomerase II activity (green box) and those compounds trying to preserve the antimitotic activity present in the natural compound (purple box).

Natural products still play a crucial role in therapy due to their interesting biopharmaceutical and pharmacological properties that make them promising lead compounds in drug discovery,^[^ ^1^, ^2^ ^]^ especially in cancer therapy. Chemomodulation of activity is one of the main strategies for the design of novel compounds based on natural products.^[^ ^3^ ^]^ This strategy allows obtaining different analogs through chemical transformations of a bioactive natural product to modulate its bioactivity and improve its pharmacological profile. Heterogenicity of tumors makes necessary to accomplish an extensively research related to chemomodulation of original structures, trying to hit a new compound with better profile and avoid resistances.^[^ ^4^ ^]^ An example of wide chemomodulation can be found in cyclolignan podophyllotoxin to improve its pharmacological profile and particularly related to its antitumoral activity.^[^ ^5^ ^]^ Podophyllotoxin is a natural compound extensively studied as cytotoxic agent extracted from *Podophyllum* spp. resin.^[^ ^6^ ^]^ Podophyllotoxin has been employed in traditional medicine as cathartic, antiviral, or antirheumatic, among other properties. Due to its systemic side effects,^[^ ^7^ ^]^ its clinical use is limited to topical formulations, for example, for the treatment of venereal warts. Among its chemical particularities, this aryltetraline‐type lignan presents several stereocenters in the tetracyclic system (Figure 1). The structure–activity relationship (SAR) of podophyllotoxin derivates has been extensively studied, and numerous chemical modifications have been carried out to chemomodulate its cytotoxic activity.^[^ ^8^ ^]^ Many podophyllotoxin derivates have been synthetized following this strategy. One of its most important derivates is the drug etoposide,^[^ ^9^ ^]^ an antitumoral drug approved by the FDA in 1983 that was included in chemotherapy protocols of hematological and solid tumors. Based on the structural modifications of etoposide, other derivatives have been synthesized. Examples are teniposide, NK‐611, GL‐311 or TOP‐53 (top panel of Figure 1).^[^ ^10^ ^]^ Not only the chemical modifications mentioned above can modify the molecular target, also the binding site within the same target can change by chemomodulation of podophyllotoxin as recently described for tubulin.^[^ ^11^ ^]^

Etoposide differs from podophyllotoxin in the *O*‐demethylation at C4′ position and the introduction of a bulky substituent in C7β instead of the C7α hydroxyl existing in the natural cyclolignan. As a consequence of these structural changes, the mechanism of cytotoxic action of etoposide changes with respect to podophyllotoxin. On the one hand, the natural product binds to the colchicine site on the β‐subunit of tubulin in the interface of α‐ and β‐ monomers, leading to the inhibition of tubulin polymerization, which results in a potent antimitotic activity.^[^ ^8^ ^]^ On the other hand, etoposide inhibits the enzyme topoisomerase II, intervening in the cell cycle at an earlier stage of replication but also blocking cell cycle at mitotic stage.^[^ ^12^ ^]^ In a general overview, the four‐ring fused system and the chiral centers (except C7 as explained above) seems to be structural requirement for the antitumoral activity of podophyllotoxin and its derivates, regardless the molecular target. Further research in podophyllotoxin derivates has retrieved other SAR conclusions.

Methylenedioxy group (A ring) (Figure 1) present in podophyllotoxin‐derived compounds seems to play an important role in cytotoxicity.^[^ ^13^ ^]^ Modifications at this ring resulted in compounds that did not improve their antitumoral properties.^[^ ^13^ ^]^ Modifications in C‐ring, for example leading to its aromatization, resulted in the loss of the E‐ring axiality, causing a significant decrease in antimitotic activity.^[^ ^14^ ^]^ The D‐ring present in the natural compound as a *trans*‐γ‐lactone has also been a crucial structural requirement for the cytotoxic activity and has remained unmodified in most of the synthesized cyclolignan derivatives, both for antimitotic and antitopoisomerase II inhibitors^[^ ^5^ ^]^ (Figure 1). Interestingly, picropodophyllin (*cis*‐lactone derivate) has been recently reported in several studies for their antitumoral properties.^[^ ^15^, ^16^ ^]^ On the other side, our research group has been involved in the design and synthesis of new podophyllotoxin derivatives lacking the γ‐lactone ring.^[^ ^17^ ^]^ The structural modifications at the C9 and C9’ positions resulting from the lactone opening has yielded several potent cytotoxic compounds that preserved the antimitotic effect and improved selectivity on solid tumor cell lines,^[^ ^18^ ^]^ as happens with our hit compound podophyllic aldehyde (Figure 1).^[^ ^17^ ^]^ This is a nonlactone cyclolignan presenting an α,β‐unsaturated aldehyde group at C9 and a methyl ester group at C9’.^[^ ^17^, ^18^, ^19^ ^]^ The methodology for obtaining analogs of podophyllic aldehyde has enabled further functionalization of cyclolignans, resulting in a library of compounds with different substitutions at positions C9 and C9’. Among these compounds, hybrid families by conjugation with other bioactive natural products^[^ ^20^, ^21^, ^22^, ^23^ ^]^ were also included.

The potential of the γ‐lactone to influence the cytotoxicity of the final compounds, together with the structural requirements for topoisomerase II inhibition, were the goals of this study. Here, we propose changes in lactone ring requirements (such as *cis*‐lactone) and the synthesis of novel nonlactonic cyclolignans based on podophyllic aldehyde (lacking γ‐lactone) and including structural features of etoposide is performed to explore the combination of tubulin‐topoisomerase II inhibition in podophyllotoxin‐related cyclolignans.

## 2 RESULTS AND DISCUSSION

### 2.1 Chemistry

**Figure 2:**
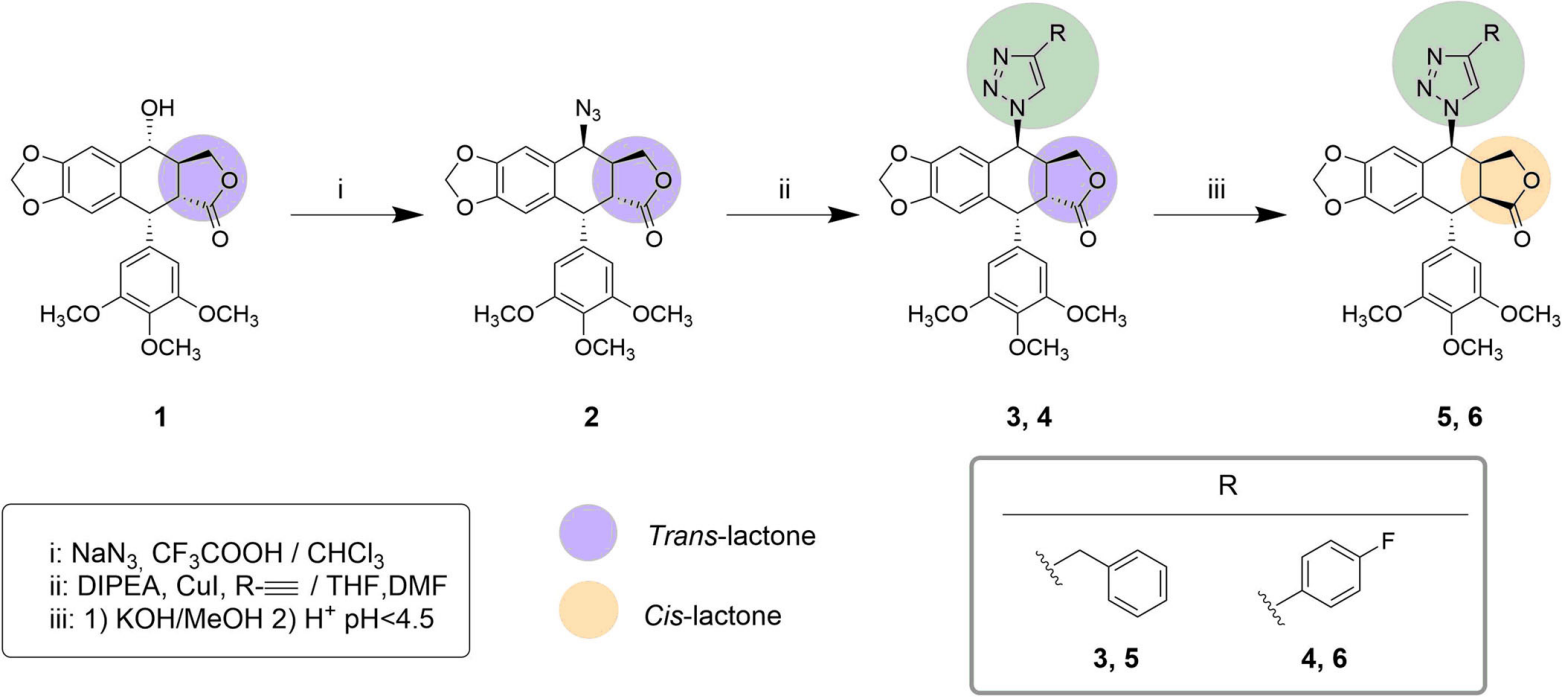
Synthetic route to obtain podophyllotoxin derivates with a C7β susbtituent and trasformation of *trans*‐lactone (purple) into the *cis*‐lactone (yellow).

Lactone ring is one of the main structural features of podophyllotoxin and its derivates, and it is preserved in etoposide and analogs. In this study, we studied whether the *trans*‐lactone moiety is essential for cytotoxic activity, as it was previously assumed, or if its modification could result in modulation of the cytotoxic activity. To this aim, picropodophyllin (*cis*‐lactone) derivates and nonlactone derivates were synthesized. They include a C7β substituent mimicking etoposide structural requirement. The first step in the chemical strategy was the introduction of different substituents at C7β position (Figure 2). Podophyllotoxin, **1**, was treated with sodium azide and trifluoroacetic acid to obtain the 7β‐azide (**2**) with an excellent yield (92%). The azide group is a very versatile functional group used in click chemistry synthetic methodology that is suitable for reaction with alkynes through a 1,3‐dipolar cycloaddition reaction.^[^ ^24^ ^]^ Maintaining *trans*‐lactone ring, azide was treated with differently substituted ethynyl derivates obtaining cyclolignans **3** and **4**. Next step was the transformation of *trans*‐lactone ring into the *cis*‐lactone. Treatment of compounds **3** and **4** in basic conditions resulted in the opening of *trans*‐lactone that easily evolves toward the *cis*‐derivates. This relactonization process yielded the *cis*‐lactone cyclolignans **5** and **6**.^[^ ^25^ ^]^

**Figure 3:**
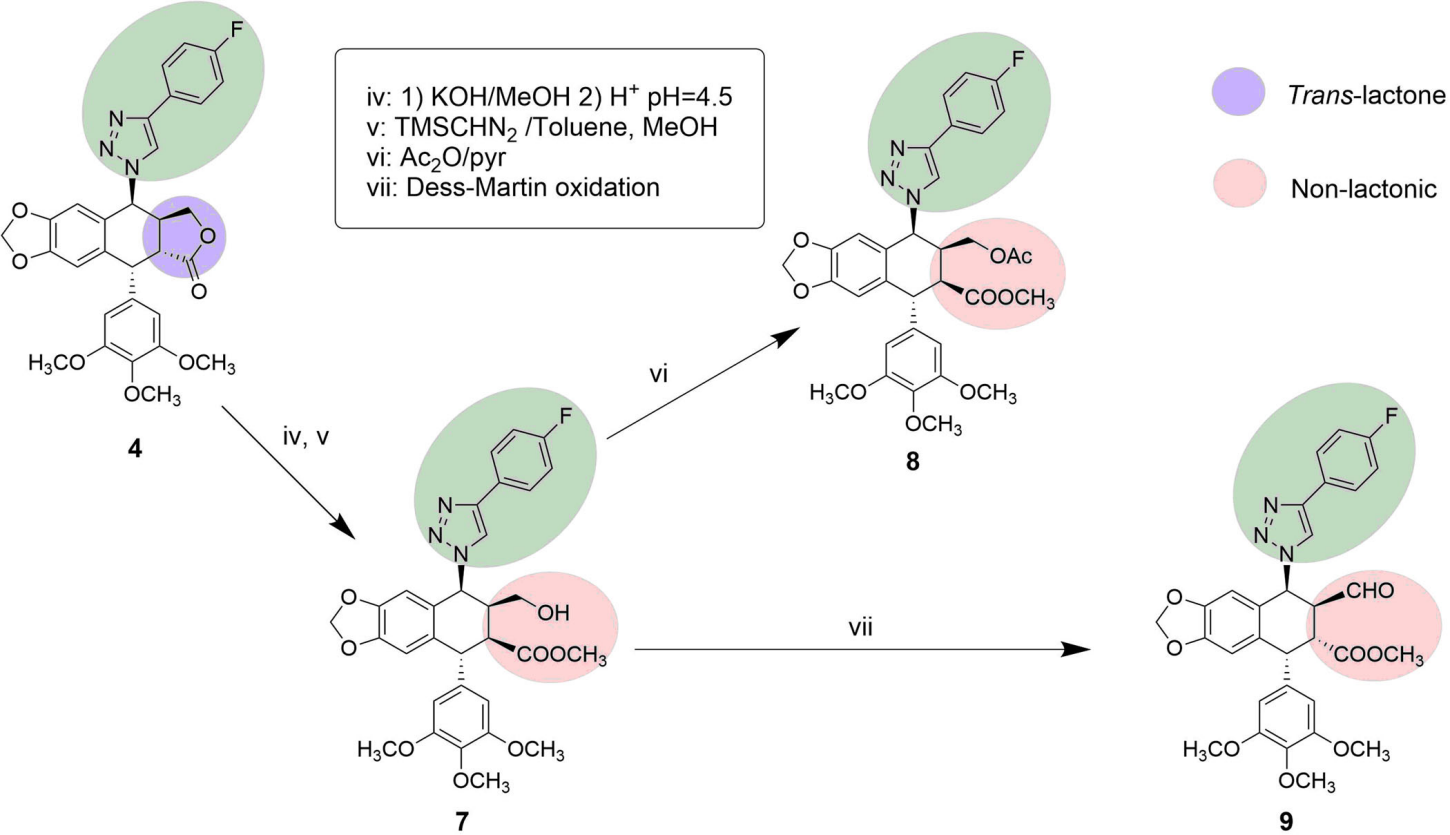
Synthetic route to obtain the podophyllotoxin derivatives **7‐9** with a substituent in C7 and lacking the γ‐lactone.

On its part, the research group has also extensive experience in opening γ‐lactone and avoiding relactonization.^[^ ^20^, ^23^ ^]^ A precise pH control (pH = 4.5) when acidulation with aqueous HCl is done after opening *trans*‐lactone in basic conditions, provides the corresponding picropodophyllic acid.^[^ ^17^ ^]^ In this study, derivative **4** was treated under these conditions followed by treatment with TMSCHN_2_ yielding the corresponding methyl ester **7** (Figure 3). This transformation, along with favoring the stability of the open lactone, provides one of the structural features of the podophyllic aldehyde analog (Figure 1). With hydroxyester **7**, two transformations were carried out at C9 position of cyclolignan to evaluate the influence of these modifications on the final cytotoxicity of the compounds.

Enhancing electrophilic character of C9 position yielded better results in previous structural‐cytotoxic studies.^[^ ^17^ ^]^ Taking as a starting point the alcohol derived from the opened‐lactone, compounds 8 and 9 were obtained, presenting at C9 position an acetoxy group and an aldehyde group, respectively. Notably, compound **9** has the structural features of podophyllic aldehyde (which inhibits tubulin polimerization) and also has a bulky substituent at position C7β as described in the literature for topoisomerase II cyclolignan inhibitors.

### 2.2 Biological assays

First, we explored cytotoxicity of cyclolignans **3**‐**9** in two different tumor cell lines, Jurkat cell line as an example of hematological neoplasia (T‐lymphocyte) and HT‐29 cell line for solid tumor (colon). Cells were tested at 72 h of drug exposure time to evaluate differences in cytotoxicity. The viability results (IC_50_ values) found for the synthesized compounds and the positive controls, podophyllic aldehyde and etoposide are shown in Table 1. At a glance, it can be seen that all compounds are more cytotoxic in the HT‐29 solid tumor line, compared with the Jurkat hematological line. Further analysis of the particular characteristics of the lactone can be found in Table 1. Regarding the influence of the presence of the *trans*‐lactone (as in the natural product podophyllotoxin and the drug etoposide) and *cis*‐lactone (picropodophyllin derivatives) arrangement, no remarkable differences in cytotoxicity was observed. Both pairs of compounds **3** and **5** (benzyl derivatives) and **4** and **6** (fluorophenyl) showed no variation in their cytotoxicity level when comparing both γ‐lactone displays. Comparing the cytotoxicity of compounds **3**‐**6** between the tested lines, all compounds exhibited cytotoxicity at the micromolar level, irrespective of their structure and the cell line tested. The cytotoxicity values obtained for compounds lacking γ‐lactone were more interesting, both among the cell lines studied and in their structural characteristics. We observed that lactone opening improves cytotoxicity in the HT‐29 cell line, where values below the micromolar level are obtained for compounds **7** and **9**, the latter reaching the cytotoxicity level of the reference compound podophyllic aldehyde and improving the etoposide value. As for the different substituents, it seems that the acetoxy group (compound **8**) in C9 position does not improve the activity neither in the Jurkat line nor in the HT‐29 line, where the lowest cytotoxicity value of the whole series of compounds studied was obtained.

**Table 1:** Cytotoxicity values (IC_50_, µM) of compounds **3‐9** and reference compounds (podophyllic aldehyde and etoposide).

Compound	Structural features		Jurkat (T cell)	HT‐29 (colon)
	R	D‐Ring		
**3**	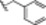	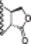	8.59 ± 1.92	5.79 ± 0.17
**5**		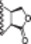	11.78 ± 1.62	4.85 ± 0.19
**4**	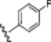	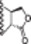	8.97 ± 2.28	3.00 ± 0.26
**6**		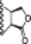	6.05 ± 2.73	1.25 ± 0.41
**7**		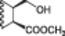	8.77 ± 0.27	0.94 ± 0.33
**8**		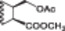	14.28 ± 1.74	1.36 ± 0.24
**9**		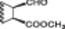	8.97 ± 1.09	**0.04 ± 0.01**
	Podophyllic aldehyde		5.23 ± 0.17	**0.028 ± 0.0061**
	Etoposide		7.09 ± 0.19	1.25 ± 0.023

**Figure 4:**
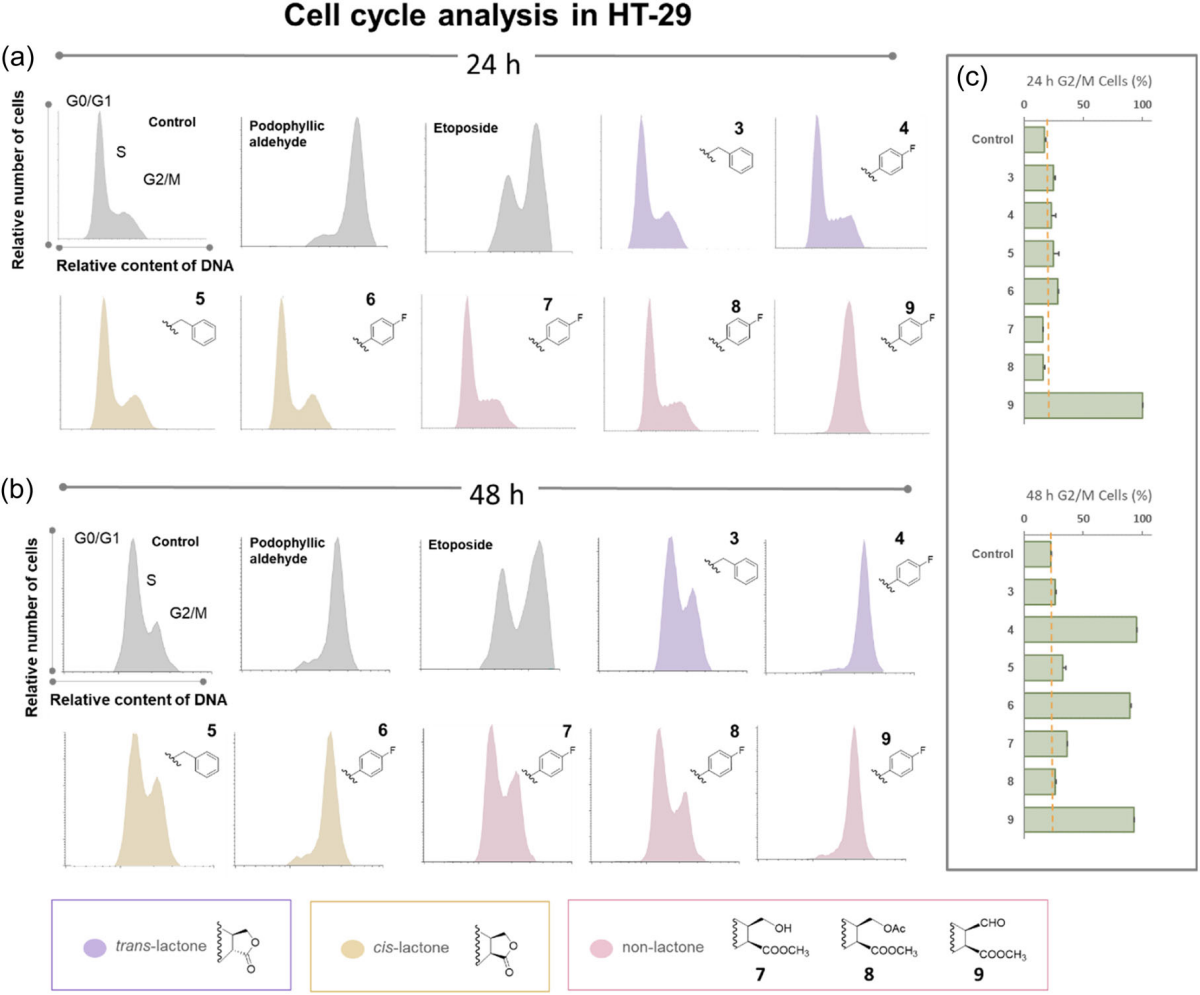
Cell cycle results for compounds **3‐9** and positive controls (podophyllic aldehyde and etoposide) at 1 µM at 24 h (a) and 48 h (b) of incubation with HT‐29 cells. Structural characteristics of compounds are also depicted in different colors. (c) Represents the value (%) of cells in G2/M phase for novel compounds tested. Orange line as cut off using control % as a reference value.

Analysis of DNA content was performed in tumor cell lines tested. Compounds included in cytotoxic assays were tested at 1 µM. Figure 4 for HT‐29 and Supporting Information S2: Figure S1 for Jurkat present the cell cycle profiles and the corresponding cell cycle phases (G0/G1, S and G2/M). In the case of Jurkat cell line at 24 h of incubation, only etoposide resulted in a cell cycle arrest (Supporting Information S2: Figure S1). The other compounds, including podophyllic aldehyde, presented no cell cycle activity in the hematological cell line.

Different and more interesting results were found for the HT‐29 line. Both positive controls showed partial (etoposide) or complete (podophyllic aldehyde) arrest in cell cycle at 24 h of incubation. At this incubation time, a complete block of the cell cycle in G2/M by compound **9** with C7β substitution and lacking the lactone group could be observed. This fact highlights the importance of the aldehyde group at C9 position for antimitotic activity, as it has been reported in previous study for podophyllic aldehyde.^[^ ^23^ ^]^ For the rest of the compounds, only a slight disturbance in the G2/M phase was observed, which increased its value with respect to the control in compounds **3**‐**6**.

To further explore the cell cycle process, the procedure was studied at a longer incubation time (48 h), since in previous studies very interesting SAR results had been obtained when the incubation time was increased.^[^ ^23^ ^]^ As a result of the study, it was observed that the positive controls maintained their cell cycle inhibition profile and that compounds **4** and **6** at 48 h of incubation showed a cell cycle inhibition profile similar to that of podophyllic aldehyde. Compound **9** also maintained the ability to block the cell cycle completely. The three compounds that inhibited cell cycle had the same substitution, 4‐(4‐fluorophenyl)triazole at the C7β position. In contrast, compounds **7** and **8**, which also included the same scaffold at position C7β, only exhibited a slight cell cycle inhibition.

### 2.3 Molecular docking

Docking and MD calculations were carried out to elucidate how compounds with better cell cycle profile interact with tubulin and topoisomerase II: **4** (*trans*‐lactone), **6** (*cis*‐lactone), and **9** (C9 aldehyde).

Regarding the interaction with tubulin, docking studies predict that compounds **4**, and **6** fit well in the colchicine binding site, where podophyllotoxin was resolved in the crystal structure of the podophyllotoxin‐tubulin complex. However, compound **9** was predicted to lie in the interface between the two protomers, close but not inside the colchicine binding site. Attempts of docking compound **9** in the colchicine binding site resulted in significantly smaller binding affinities. The position of the three ligands in the binding site remain stable within the 100 ns MD simulation. Binding energies and the interaction between the two protomers along the MD simulation were calculated to evaluate the potential of the compounds, as tubulin polymerization inhibitors. All three compounds showed increased binding affinity to tubulin with respect to podophyllic aldehyde (Table 2), and the highest affinity was obtained for the *cis*‐lactone compound (**6**). Regarding the interaction between α and β tubulin, docking of the three compounds lead to a slightly increased interaction energy compared with podophyllic aldehyde (although within the standard deviation of the data). It was previously speculated^[^ ^21^ ^]^ that the binding of podophyllotoxin inhibits tubulin dimerization by decreasing the protomer–protomer affinity, so we predict that compounds **4**, **6**, and **9** should not inhibit tubulin dimerization more efficiently than podophyllic aldehyde taken as reference in this study.

**Table 2:** Docking energies (kcal/mol) and protomer–protomer interaction energies (kcal/mol) calculated for podophyllic aldehyde and compounds **4**, **6**, and **9** for tubulin interaction.

Compound	docking Δ*G*/(kcal/mol)	MM/GBSAΔG/(kcal/mol)	Protomers Interaction/(kcal/mol)
Podophyllic aldehyde	−8.5^a^	–	−450 ± 80^a^
**4**	−11.0	−31 ± 4	−520 ± 100
**6**	−12.4	−41 ± 4	−510 ± 90
**9**	−10.6	−31 ± 4	−500 ± 120

^a^ Results for podophyllic aldehyde were extracted from Hernández et al.^[^ ^21^ ^]^

**Figure 5:**
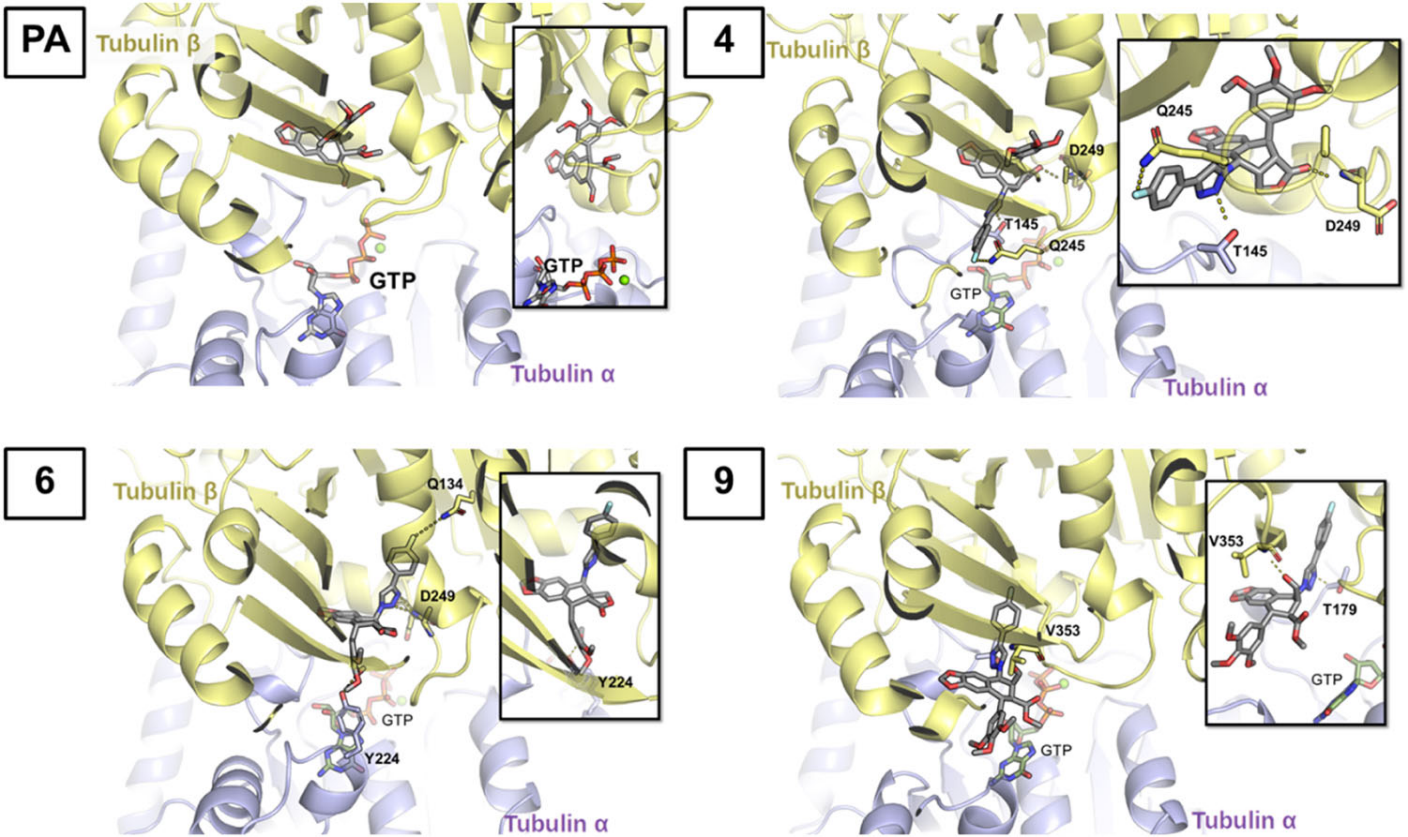
Docking complexes of compounds in the colchicine site of tubulin, podophyllic aldehyde (PA), **4**, **6**, and **9**. Inhibitors, GTP, and those residues that interact with the inhibitors via hydrogen bonds are shown atomistically. The two protomers, tubulin α and β, are shown in purple and yellow, respectively.

To get further insight for the binding between cyclolignans and tubulin strands, the interaction with the target is depicted in Figure 5. Podophyllic aldehyde fits in the β‐tubulin strand placing the trimethoxyphenyl residue in the inner part of the pocket while the four fused rings are located closer to the interface of the two protomers (Figure 5). Compound **4** presenting the *trans*‐lactone is placed in a similar disposition, maintaining the trimethoxyphenyl ring in the β‐tubulin pocket and the aryltretraline lactone in the interface between the two protomers. In particular, the carbonyl group of the lactone interacts with the residue Asp‐249. Such interaction with this residue was also observed by us in podophyllotoxin MD studies previously reported,^[^ ^23^ ^]^ what may suggest a similar behavior of both cyclolignans containing the same configuration of γ‐lactone in tubulin interaction. Moreover, **4** also interacts with another residue of β‐strand (Glu‐245) with the fluor substituent and interact with α‐tubulin thanks to one of the nitrogen atoms in the triazole ring. The other compounds, **6** and **9**, presented a different disposition in β‐tubulin pocket, arranged with the C7 substituent on the pocket where the trimethoxyphenyl ring was placed in the previous cases. Compound **6** places further inside the pocket, interacting with the β‐tubulin residue Gln‐134. Despite the inverse disposition, the triazole group also plays an important role for the interaction with β‐tubulin as one nitrogen also interacts with the same residue as compound **4** (Asp‐249). This compound, as previous one, also interacts with α‐tubulin, placing itself in the interface of the protomers, through an interaction with Tyr‐224. Compound **9**, with aldehyde group, interacts with β‐strand (Val‐353) through its carbonyl group and is also placed in the interface of the protomers but not inside the colchicine site as podophyllic aldehyde. In this case, triazole ring interact with α‐strand in the residue Tyr‐179. In the three new compounds, the interaction with both tubulin strands is higher than in podophyllic aldehyde as different H‐bonds have been detected, what can justify the increase in binding energies respect to the initial compound. Binding free energies were calculated through docking and MM/GBSA method. Leaving aside the difference in the absolute values between both methods, which are not relevant, higher binding free energies (in absolute value) are obtained for compound **6**. Regarding the absolute values of the MM/GBSA binding energies, they are in line with the best MM/GBSA binding energies reported in reference^[^ ^26^ ^]^ for the study of amine and acetyl terminated PAMAM dendrimers, and are lower than those calculated in reference^[^ ^11^ ^]^ for the binding of podophyllotoxin to tubulin.

Regarding the potential of **4**, **6**, and **9** to inhibit DNA‐topoisomerase IIα, compounds **4** and **6** showed very high binding affinities, slightly higher than etoposide that is considered as a reference. On in its part, binding of **9** resulted in a significantly lower affinity. When we calculated the ligand–protein and ligand–DNA interaction energy in the ternary complex, we consistently found that the interaction with DNA is stronger than interaction with the protein (Table 3).

**Table 3:** Docking energies (kcal/mol) calculated for etoposide and compounds **4**, **6**, and **9** for topoisomerase II interaction.

Compound	Docking	MM/GBSA	Ligand–protein/(kcal/mol)	Ligand–DNA/(kcal/mol)
	Δ*G*/(kcal/mol)	Δ*G*/(kcal/mol)		
**Etoposide**	−12.4^a^	–	−37 ± 10^a^	−54 ± 10^a^
4	−12.9	−37 ± 3	−32 ± 8	−59 ± 7
6	−13.0	−32 ± 4	−25 ± 8	−56 ± 6
9	−9.5	−32 ± 4	−31 ± 8	−69 ± 8

*Note*: Ligand–protein and Ligand–DNA interaction energies are also calculated.

^a^ Results for the etoposide were extracted from Hernández et al.^[^ ^21^ ^]^

**Figure 6:**
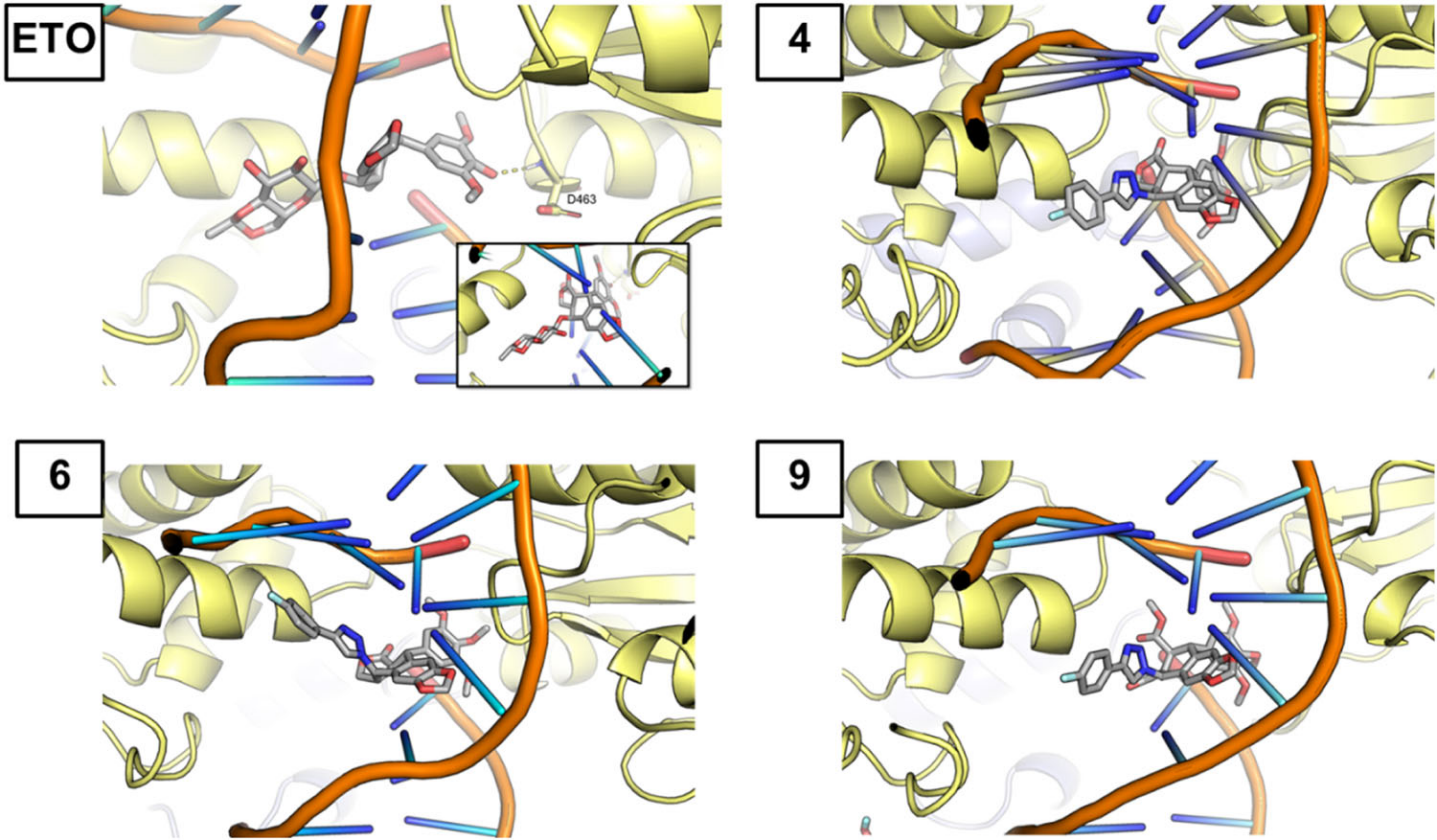
Docking complexes of compounds etoposide (ETO), **4**, **6**, and **9**. Inhibitors and residue that interact with them via hydrogen bonds are shown atomistically.

The structure of the protein–DNA–ligand complexes after MD simulations is shown in Figure 6. Taking etoposide as the reference for topoisomerase II inhibition, it can be observed that the ligand is intercalated between two DNA basis pairs. The hydroxyl group at C4’ ring interacts with the backbone of Asp‐463. This interaction seems to be responsible for the higher interaction between etoposide and the protein, which is higher than that obtained for the other three ligands that had a methoxy group at C4’. The position of **4**, **6**, and **9** is similar, all of them intercalated between the DNA strands, with their trimethoxyphenyl group mimicking the disposition showed for etoposide. Calculations predict high binding energies for all the compounds. In the present case while docking binding energies are similar for compounds **4** and **6**, MM/GBSA calculations predict a higher binding energy for **4** (in absolute value).

## 3 DISCUSSION

Chemomodulation of natural products remains a very successful strategy in the search for new antitumor compounds. The unique structural complexity of the natural compounds enables modification of some of their molecular moieties leading to new derivates with modified, and sometimes enhanced, activities. In this study, we have used this approach to stablish an optimal route to obtain compounds that meet the structural requirements for binding to two target antitumor proteins, tubulin and topoisomerase II.^[^ ^27^, ^28^ ^]^ Synthetically this has been a challenge. First, relactonization of the D‐ring when trying to obtain the nonlactonic cyclolignans is a process that highly favored the *cis*‐lactone formation.^[^ ^18^ ^]^ In our previous studies, the presence of the hydroxyl group at the C7 position^[^ ^20^, ^23^ ^]^ enabled the protection of the dihydroxyacid resulting from the lactone opening in the form of an acetonide. However, this is not possible if a bulky substituent must be incorporated at the C7 position, which has been proved necessary for the antitopoisomerase II activity.

In this study, we have synthetized compounds **7**‐**9**, differentially substituted at C9 and C9’ positions. To fulfill the structural requirements, it would still be necessary further structural features of podophyllic aldehyde and etoposide, namely the presence of an unsaturation in C7 position to have the conjugated aldehyde as well as the *O*‐demethylation at the C4’ position. Undoubtedly, the very satisfactory results obtained in this study encourage us to extend this family of compounds by including these features. Following the same direction on how to increase the number of compounds, an approach could come from the inclusion of other bulky substituents at C9’ position, a strategy that has proved very successful in previous studies, while maintaining α,β‐unsaturated aldehyde at the C9 position.^[^ ^18^ ^]^

With regard to the cytotoxicity and cell cycle inhibition described in this study, the difference in the activity of the synthesized compounds between the solid cancer tumor line and the hematological cancer tumor line is of particular relevance. Strikingly, some of the compounds showed comparable cytotoxicity between the two cell lines (both in the micromolar range) with differences in activity in the cell cycle, where no cell cycle arrest was found in Jurkat cell line. This is in agreement with previous results,^[^ ^18^ ^]^ where some compounds that were found to be cytotoxic did not show cell cycle inhibition allowing other mechanisms or targets to be explored.

Dynamic molecular studies have provided further remarks about the SAR of the compounds proposed in this study, which can explain the total blockade of the cell cycle observed in the DNA content assay for the three compounds with the 4‐fluorophenyl substituent. Our results suggest that they bind more effectively than podophyllic aldehyde to the tubulin, although their binding slightly increases the interaction between the alpha and beta subunits. Therefore, in view of these results, their cycle inhibition effect can be attributed to this antitubulin mechanism of action. Regarding the interaction with topoisomerase II and as expected, compounds **4** and **6**, have also been shown to have the ability to successfully interact with the broken DNA strand in the complex with topoisomerase II. Compound **9**, which is the result of the cleavage of the γ‐lactone and further functionalization to aldehyde at C9, maintains the arrangement and orientation of the molecule in the DNA break, suggesting that the activity of this molecule may be due to the interaction not only with tubulin, but also with topoisomerase II.

The binding affinity of compound **9** to tubulin and topoisomerase II is smaller than for the other compounds. Despite that, compound **9** reached the best cytotoxic values in HT‐29. It should be noticed however that MD simulations revealed that (a) **9** interacts stronger with DNA strand in the topoisomerase‐II complex than any of the other ligands and (b) binding of **9** to tubulin disturbed the interaction between the two tubulin protomers, as revealed by the slightly smaller interaction energy between the two protomers. This fact could be in accordance with the possible existence of a third mechanism of action suggested for cyclolignans^[^ ^18^ ^]^ and not yet elucidated. Also noteworthy from in silico studies is the role that *O*‐demethylation at C4’ appears to play in the interaction of etoposide with topoisomerase II. In view of the results obtained for compound **9**, the inclusion of this modification could also be the key to improve the interaction with topoisomerase II and accomplish the duality in the interaction with the proposed targets.

## 4 CONCLUSION

Overall, in this study, a new synthetic approach to yield podophyllotoxin cyclolignan‐derived compounds with potential dual molecular activity requirements (antimitotic and antitopoisomerase II) has been achieved. In the case of cytotoxicity, compound **9** reached the activity of podophyllic aldehyde and also presented the ability to inhibit the cell cycle. Molecular docking studies have revealed interesting properties for this compound to bind tubulin and topoisomerase II, although the interaction with the latter did not reach the etoposide level. New molecular characteristics using C7 substituents and modifications in γ‐lactone ring open the door to the design and synthesis of new and differentially functionalized cyclolignans that might be able to improve binding to the two targets. As a whole, these results encourage further work on the chemomodulation of natural compounds, especially in the research of new podophyllotoxin derivatives with improved therapeutic perspectives. Based in our results and taking into consideration this broad spectrum of activities, it should be important to consider the *cis*‐lactone configuration for further studies, both antimitotic effect and topoisomerase II inhibition. It would also allow to repurpose our compound for other targets and pharmacological activities.

## 5 EXPERIMENTAL

### 5.1 Chemistry

#### 5.1.1 General

^1^H and ^13^C NMR experiments were recorded on Varian Mercury Vx (400 MHz) or Bruker Advance NEO 400 MHz spectrometers in CDCl_3_ using the residual solvent signal as reference. ^1^H‐NMR and ^13^C‐NMR are displayed in Supporting Information S2: Figures S2–S8. Chemical shift (δ) values are expressed in ppm, followed by multiplicity, and coupling constants (*J*) in Hz. High‐Resolution Mass Spectrometry (HRMS) was run on a Thermo Orbitrap QExactive Focus QSTAR XL using electrospray ionization (ES) at 5500 V with a Thermo Vanish chromatograph. Solvents and reagents were purified by standard procedures as necessary. Dimethylformamide (DMF) and dichloromethane (DCM) were dried over molecular sieves and tetrahydrofuran (THF) was dried over sodium. Column chromatography (CC) purifications were carried out using silica gel 60 (0.040–0.063 mm, 230–400 mesh, Merck).

The starting material podophyllotoxin (**1**) was isolated from commercial resin of *Podophyllum emodi*, following the protocol previously described by our research group.^[^ ^13^ ^]^ Podophyllic aldehyde was synthesized as described in reference.^[^ ^17^ ^]^ Etoposide was purchased from Sigma Aldrich (#E1383).

The InChI codes of the investigated compounds, together with some biological activity data, are provided as Supporting Information.

#### 5.1.2 Procedure for the synthesis of (5*R*,5a*R*,8a*S*,9*S*)−9‐azido‐5‐(3,4,5‐trimethoxyphenyl)−5,8,8a,9‐tetrahydrofuro[3’,4’:6,7]naphtho[2,3‐*d*][1,3]dioxol‐6(5a*H*)‐one (**2**)

Podophyllotoxin (**1**, 450 mg, 1.09 mmol), and sodium azide (191 mg, 2.93 mmol) were dissolved in chloroform. Inert atmosphere was made and then, trifluoroacetic acid (1.1 mL, 15 mmol) was added. The reaction mixture was stirred at room temperature overnight; then, it was quenched with NaHCO_3_ and extracted with CHCl_3_. The organic layer was washed with saturated aqueous solution of NaCl, dried over Na_2_SO_4_, and filtered. Concentration under reduced pressure yielded compound **2** (416 mg, 1.00 mmol, 92%). ^1^H‐NMR (CDCl_3_): *δ* 6.74 (*s*, 1H, H6), 6.52 (*s*, 1H, H3), 6.19 (*s*, 2H, H2’,H6’), 5.97 (*d*, 1H, *J* = 1.4 Hz, H10), 5.94 (*d*, 1H, *J* = 1.4 Hz, H10), 4.70 (*d*, 1H, *J* = 3.6 Hz, H7), 4.56 (*d*, 1H, *J* = 5.6 Hz, H7’), 4.25 (*dd*, 2H, *J* = 2.4, 9.2 Hz, H9), 3.73 (*s*, 3H, H11’), 3.69 (*s*, 6H, H10’, H12’), 3.10 (*dd*, 1H, H8’). ^13^C‐NMR: Supporting Information S2: Table S1.

#### 5.1.3 General procedure for azide‐alkyne Huisgen 1,3‐dipolar cycloadditions

To a stirring solution of the propargyl derivative (3.5 equiv.) in 1 mL of dry DMF and 1 mL of dry THF, CuI (0.2 equiv.) and *N,N*‐diisopropylethylamine (DIPEA) (13 equiv.) were added. Then the azide derivative 2 (1 equiv.) was added. The mixture was stirred overnight at room temperature under inert atmosphere. Then, the reaction mixture was quenched with water and crude was extracted with EtOAc. The organic layer was washed with saturated aqueous NaCl, dried over Na_2_SO_4_ and concentrated in vacuum. Purification through CC on silica gel yielded the final products.

(5*R*,5a*R*,8a*S*,9*S*)−9‐(4‐Benzyl‐1*H*−1,2,3‐triazol‐1‐yl)−5‐(3,4,5‐trimethoxyphenyl)−5,8,8a,9‐tetrahydrofuro[3’,4’:6,7]naphtho[2,3‐*d*][1,3]dioxol‐6(5a*H*)‐one (**3**): Following the general method for Huisgen 1,3‐dipolar cycloadditions, 217 µL (1.7 mmol) of 3‐phenylprop‐1‐yne were dissolved in 1 mL of dry DMF and 1 mL of dry THF. Then, CuI (23 mg, 0.12 mmol), DIPEA (1.5 mL, 23 mmol) and compound **2** (245 mg, 0.582 mmol) were added. The crude was purified through CC on silica gel, eluting with DCM/EtOAc 90:10 to yield compound **3** (190 mg, 0.342 mmol, 58%). ^1^H‐NMR (CDCl_3_): *δ* 6.92 (*s*, 1H, H11), 6.61 (*s*, 1H, H6), 6.58 (*s*, 1H, H3), 6.43 (*s*, 2H, H2’, H6’), 6.02 (*d*, 1H, H7), 6.00 (*d*, 1H, *J* = 1.4 Hz, H10), 5.98 (*d*, 1H, *J* = 1.4 Hz, H10), 4.70 (*d*, 1H, H7’), 4.07–4.33 (*m*, 2H, H9), 4.07 (*m*, 1H, H8), 3.82 (*s*, 3H, H11’), 3.76 (*s*, 6H, H10’, H12’), 3.18 (*m*, 1H, H9), 2.93 (*dd*, 1H, H8’). Benzyl substituent: *δ* 7.22–7.33 (*m*, 5H, ‐CH_2_‐C_6_H_5_), 4.04 (*s*, 2H, ‐CH_2_‐C_6_H_5_). ^13^C‐NMR: Supporting Information S2: Table S1. HRMS: calculated for [C_31_H_29_N_3_O_7_+H^+^] 556.2078 u; found 556.2078 m/z.

(5*R*,5a*R*,8a*S*,9*S*)−9‐[4‐(4‐Fluorophenyl)−1*H*−1,2,3‐triazol‐1‐yl]−5‐(3,4,5‐trimethoxyphenyl)−5,8,8a,9‐tetrahydrofuro[3’,4’:6,7]naphtho[2,3‐*d*][1,3]dioxol‐6(5a*H*)‐one (**4**): Following the general method for Huisgen 1,3‐ dipolar cycloadditions, 175 µL (1.37 mmol) of 1‐ethynyl‐4‐fluorobenzene were dissolved in 1 mL of dry DMF and 1 mL of dry THF and subsequently CuI (18 mg, 0.09 mmol), DIPEA (1.1 mL, 18.4 mmol) and compound **2** (205 mg, 0.581 mmol) were added. The crude was purified through CC on silica gel, eluting with DCM/EtOAc 90:10 to yield compound **4** (185 mg 0.410 mmol, 72%). ^1^H‐NMR (CDCl_3_): *δ* 7.36 (*s*,1H, H11), 6.67 (*s*, 1H, H6), 6.66 (*s*, 1H, H3), 6.33 (*s*, 2H, H2’,H6’), 6.14 (*d*, 1H, *J* = 5.2 Hz, H7), 6.01 (*d*, 1H, H10), 6.00 (*d*, 1H, H10), 4.76 (*d*, 1H, H7’), 4.43 (*m*, 1H, H9), 3.78 (*s*, 3H, H11’), 3.77 (*s*, 6H, H10’,H12’), 3.37 (*m*, 1H, H9), 3.24 (*m*, 1H, H8), 3.10 (*dd*, 1H, *J* = 5.2, 14.4 Hz, H8’). Fluorophenyl substituent: 7.75 (*m*), 7.11 (*m*). ^13^C‐NMR: Supporting Information S2: Table S1. HRMS: calculated for [C_30_H_26_FN_3_O_7_+ H^+^] 560.1823 u; found 560.1828 m/z.

#### 5.1.4 General procedure for *cis*‐lactone cyclolignans

Corresponding cyclolignans were dissolved in methanol (5 mL) and a solution of KOH 5% (15 mL) in methanol was added. The mixture was stirred during 30 min at room temperature and then HCl 2 N was added until pH = 3 was reached. Then, solvent was eliminated through vacuum and the aqueous phase was extracted with EtOAc. Subsequently, the organic layer was washed with saturated aqueous solution of NaCl, dried over Na_2_SO_4_ and evaporated to obtain the *cis*‐lactone derivatives.

5.1.5

(5*R*,5a*S*,8a*S*,9*S*)−9‐(4‐Benzyl‐1*H*−1,2,3‐triazol‐1‐yl)−5‐(3,4,5‐trimethoxyphenyl)−5,8,8a,9‐tetrahydrofuro[3’,4’:6,7]naphtho[2,3‐*d*][1,3]dioxol‐6(5a*H*)‐one (**5**): From compound **3** (222 mg, 0.40 mmol) following the general procedure for *cis*‐lactone cyclolignans, obtaining **5** (67 mg, 0.12 mmol, 30%). ^1^H‐NMR (CDCl_3_): *δ* 7.05 (*s*, 1H, H11), 6.72 (*s*, 1H, H6), 6.32 (*s*, 2H, H2’, H6’), 6.13 (*s*, 1H, H3), 5.99 (*d*, 1H, H10), 5.98 (*d*, 1H, H10), 5.70 (*d*, 1H, H7), 4.57(*d*, 1H, H7’), 4.51 (*m*, 1H, H9), 4.40 (*m*, 1H, H9), 3.80 (*s*, 3H, H11’), 3.76 (*s*, 6H, H10’, H12’), 3.64 (*m*, 1H, H8), 3.56 (*dd*, 1H, H8’). Benzyl substituent: *δ* 7.22–7.32 (*m*, 5H, ‐CH_2_‐C_6_H_5_), 4.10, (*s*, 2H, ‐CH_2_‐C_6_H_5_) ^13^C‐NMR: Supporting Information S2: Table S1.

(5*R*,5a*S*,8a*S*,9*S*)−9‐[4‐(4‐Fluorophenyl)−1*H*−1,2,3‐triazol‐1‐yl]−5‐(3,4,5‐trimethoxyphenyl)−5,8,8a,9‐tetrahydrofuro[3’,4’:6,7]naphtho[2,3‐*d*][1,3]dioxol‐6(5a*H*)‐one (**6**): From compound **4** (94 mg, 0.18 mmol) following the general procedure for *cis*‐lactone cyclolignans, obtaining **6** (38 mg, 0.07 mmol, 37%). ^1^H‐NMR(CDCl_3_): *δ* 7.51(*s*, 1H, H11), 6.74 (*s*, 1H, H6), 6.37 (*s*, 2H, H2’, H6’), 6.26 (*s*, 1H, H3), 6.02 (*d*, 1H, J = 4.4 Hz, H7), 5.85 (*d*, 2H, *J* = 1.4 Hz, H10), 5.84 (*d*, 2H, *J* = 1.4 Hz, H10), 4.76 (*d*, 1H *J* = 4.4 Hz, H7’), 4.55 (*m*, 2H, H9), 4.43 (*m*, 2H, H9), 3.79 (*s*, 3H, H11’), 3.77 (*s*, 6H, H10’, H12’), 3.65 (*m*, 1H, H8), 3.52 (*dd*, 1H, *J* = 2.2, 10, H8’). Fluorophenyl substituent: 7.77 (*m*), 7.10 (*m*). ^13^C‐NMR: Supporting Information S2: Table S1.

#### 5.1.6 γ‐Lactone cleavage and functionalization reactions

Methyl (5*R*,6*S*,7*S*,8*S*)−8‐[4‐(4‐fluorophenyl)−1*H*−1,2,3‐triazol‐1‐yl]−7‐(hydroxymethyl)−5‐(3,4,5‐trimethoxyphenyl)−5,6,7,8‐tetrahydronaphtho[2,3‐*d*][1,3]dioxole‐6‐carboxylate (**7**): Compound **4** (149 mg, 0.27 mmol) was dissolved in 5 mL of methanol and 15 mL of KOH 5% solution in methanol were added. The mixture was stirred for 2 h at room temperature and then, HCl 2 N was added until pH = 4.5 was reached. Subsequently, solvent was eliminated through reduced pressure and aqueous phase was extracted with EtOAc. The resulting organic layer obtained was washed with saturated aqueous solution of NaCl, dried over Na_2_SO_4_ and concentrated in vacuum. The crude was redissolved in 8 mL of a solution of toluene/methanol (1:1) and 220 µL of a hexane solution (2 M) of TMSCHN_2_ were added under inert atmosphere. The mixture was stirred during 30 min at room temperature and solvent was evaporated after completion of the reaction. Crude product was purified over CC on silica gel, eluting with DCM/EtOAc 80:20, yielding compound **7** (70 mg, 0.14 mmol, 55%). ^1^H‐NMR(CDCl_3_): *δ* 7.19(*s*, 1H, H11), 6.59 (*s*, 1H, H3), 6.45 (*s*, 1H, H6), 6.12 (*d*, 1H, *J* = 4.8 Hz, H7), 6.11 (*s*, 2H, H2’, H6’), 5.94 (*d*, 1H, *J* = 1.4, H10); 5.93 (*d*, 1H, *J* = 1.4, H10), 4.35 (*m*, 1H, H9), 4.23–4.70 (*m*, 1H, H9), 4.12 (*m*, 1H, H7’), 3.77 (*s*, 3H, H11’), 3.71 (*s*, 6H, H10’, H12’), 3.21 (*s*, 3H, 9’‐OCH_3_), 2.79 (*dd*, *J* = 2.2 and 10.0, 1H, H8’), 2.25 (*m*, 1H, H8). Fluorophenyl substituent: 7.03 (*m*), 7.68 (*m*). ^13^C‐NMR: Supporting Information S2: Table S1. HRMS: Calculated for [C_31_H_30_FN_3_O_8_ +H^+^]: 592.2089 u; found: 592.2081 m/z.

Methyl (5*R*,6*S*,7*S*,8*S*)−7‐(acetoxymethyl)−8‐[4‐(4‐fluorophenyl)−1*H*−1,2,3‐triazol‐1‐yl]−5‐(3,4,5‐trimethoxyphenyl)−5,6,7,8‐tetrahydronaphtho[2,3‐*d*][1,3] dioxole‐6‐carboxylate (**8**): To a solution of compound **7** (40 mg, 0.072 mmol) in 0.5 mL of pyridine, 0.5 mL of acetic anhydride were added. The mixture was stirred at room temperature in the dark during 24 h. After completion of the reaction, ice was added, and the reaction crude was extracted with EtOAc. Then, the organic layer was washed with HCl 2 N, and subsequently with saturated aqueous solutions of NaHCO_3_ and NaCl, respectively. The organic layer was dried over N_2_SO_4_ and concentrated through vacuum. Compound **8** (27 mg, 0.042 mmol, 61%) was obtained after the purification of the crude product by CC on silica gel, using a mixture of hexane/EtOAc 50:50 as eluent. ^1^H‐NMR (CDCl_3_): *δ* 7.34 (*s*, 1H, H11), 6.56 (*s*, 1H, H6), 6.44 (*s*, 1H, H3), 5.98 (*d*, 1H, H10); 5.97 (*d*, 1H, H10), 5.24 (*s*, 1H, H7), 5.24 (*s*, 2H, H2’, H6’), 4.69 (*d*, 1H *J* = 4.8 Hz, H7’), 4.23–4.70 (*m*, 1H, H9), 3.85 (*s*, 3H, H11’), 3.84 (*s*, 6H, H10’, H12’), 3.41 (*s*, 3H, 9’‐OCH_3_), 3.09 (*m*, 1H, H8’), 3.07 (*m*, 1H, H8), 1.91 (*s*, 3H, 9‐OAC). Fluorophenyl substituent: 7.08 (*m*), 7.77 (*m*) ^13^C‐NMR: Supporting Information S2: Table S1.

Methyl (5*R*,6*R*,7*S*,8*S*)−8‐[4‐(4‐fluorophenyl)−1*H*−1,2,3‐triazol‐1‐yl]−7‐formyl‐5‐(3,4,5‐trimethoxyphenyl)−5,6,7,8‐tetrahydronaphtho[2,3‐*d*][1,3]dioxole‐6‐carboxylate (**9**): Compound **7** (29 mg, 0.051 mmol) was dissolved in 2 mL of DCM and Dess‐Martin periodinane (28 mg, 0.063 mmol) was added. The mixture was stirred at room temperature for 30 min. Subsequently, an aqueous solution of NaHCO_3_ 10% was added and the crude was extracted with DCM. The organic layer was washed with saturated aqueous solution of NaCl, dried over Na_2_SO_4_ and concentrated through vacuum, obtaining **9** (17 mg, 0.03, 56%). ^1^H‐NMR (CDCl_3_): *δ* 9.98 (*s*, 1H, 9‐CHO). 6.95 (*s*, 1H, H11), 6.56 (*s*, 1H, H6), 6.39 (*s*, 1H, H7), 6.39 (*s*, 1H, H3), 6.00 (*s*, 2H, H2’, H6’), 5.99 (*d*, *J* = 1.2, 1H, H10), 5.98 (*d*, *J* = 1.2, 1H, H10), 4.85 (*d*, 1H *J* = 4.8 Hz, H7’), 3.86 (*s*, 3H, H11’), 3.85 (*s*, 6H, H10’, H12’), 3.49 (*m*, 1H, H8’), 3.46 (*m*, 1H, H8), 3.40 (*s*, 3H, 9’‐OCH_3_). Fluorophenyl substituent: 7.10 (*m*), 7.77 (*m*). ^13^C‐NMR: Supporting Information S2: Table S1.

### 5.2 Biological assays

#### 5.2.1 Cell culture

Tumoral cell lines HT‐29 (ATCC® HTB‐38™) and Jurkat (DSMZ ACC 282) were incubated with the corresponding media (DMEM for HT‐29 and RPMI for Jurkat) supplemented with fetal bovine serum (10%) and penicillin/streptomycin (1%) at 37°C and 5% CO_2_. Cells were subcultured when confluence reached ~80%. Trypsin was used to detached HT‐29 cells. Cells where centrifugated at 1200 rpm for 5 min and resuspended in fresh medium. When necessary, cells were counted with Neubauer chambre.

#### 5.2.2 Viability assay

Evaluation of cytotoxicity was performed with colorimetric assay of MTT. Procedure for HT‐29 was optimized in previous reports.^[^ ^23^ ^]^ Also, protocol for Jurkat cell lines was performed following other previous report.^[^ ^29^ ^]^

#### 5.2.3 Cell cycle evaluation

For measurement of DNA content, Cycloscope reagent (Cytognos) was employed, using the protocol reported previously by us.^[^ ^23^ ^]^ Cells were acquired in BD Accuri 6 and analysis was performed in Infinicyt software (Cytognos).^[^ ^23^, ^29^ ^]^

### 5.3 Molecular docking

Docking and molecular dynamics (MD) simulations were carried out following the same protocol and structures as in reference.^[^ ^21^ ^]^ To sum up, models for the tubulin dimer and topoisomerase II were built based on the PDB 1SA1 and 5GWK crystal structures,^[^ ^30^ ^]^ respectively. In reference,^[^ ^21^ ^]^ we ran MD simulations with etoposide, podophyllic aldehyde, or podophyllotoxin docked to the binding site of the two proteins. We used the final structure of the simulations and the initial model systems to prepare the MD simulations carried out in this study, where **4**, **6**, and **9** were included in the binding site of the proteins.

Once the complexes were prepared, they were placed in the center of a cubic water box large enough to contain the protein and at least 10 Å of solvent on all sides. K^+^ and Cl^‐^ were added to the system to mimic the intracellular conditions. Once the system was prepared, 100 ns MD simulations at constant temperature (303.15 K) and pressure (1 bar) were run using NAMD,^[^ ^31^ ^]^ CHARMM36 force‐field,^[^ ^32^, ^33^ ^]^ and Particle Mesh Ewald method^[^ ^34^ ^]^ to account for the electrostatics of the periodic boundary conditions. Parameters for **4**, **6**, and **9** were assigned using ParamChem.^[^ ^35^ ^]^ A 2 fs time step and the ShakeH^[^ ^36^ ^]^ algorithm were used. All structures were stable throughout the 100 ns of MD simulations, and all ligands remained in their pocket. The RMSD of the protein along the MD simulations is shown in Figure S9 as in reference^[^ ^21^ ^]^ a small restrained between the DNA strands was added to the topoisomerase II simulations to avoid DNA breakage throughout the simulations.

To calculate the docking energy corresponding to each of the ligands, the coordinates of the protein and DNA chains of last frames of the MD simulations were extracted for a subsequent docking stage, and carried out using AutoDock Vina.^[^ ^37^, ^38^ ^]^ To get some insight into the differences among the affinities between the ligands and proteins, interaction energies were calculated throughout the MD trajectories using NAMD.

Fifty structures corresponding to the last 50 ns of the simulation were analyzed to calculate MM‐GBSA^[^ ^39^ ^]^ ligand binding energies using NAMD.^[^ ^31^ ^]^ In these calculations, the explicit water molecules and solvent ions were removed, and the MM‐GBSA analysis was performed on three subsets of the system: (a) the ligand alone, (b) the receptor (formed by the protein, ATP, or DNA strands), and (c) the complex between the ligand and the receptor. Δ*G*_bind_ was calculated as:

(1) $${\Delta G}_{\mathrm{bind}}=G_{\mathrm{complex}}-G_{\mathrm{repector}}{-\;G}_{\mathrm{ligand}},$$

where each of the free energies has contributions of the internal, electrostatic, van der Waals energies, and contributions to the solvation free energies. Conformational entropy change is expected to be negligible,^[^ ^39^ ^]^ and was neglected in the calculations as in references.^[^ ^26^, ^40^ ^]^

## CONFLICT OF INTEREST STATEMENT

The authors declare no conflict of interest.

## Supporting information

Supporting information.

Supporting information.

## Data Availability

The data that support the findings of this study are available from the corresponding author upon reasonable request.
