# Supplementary material for: Insights into podophyllotoxin lactone features: New cyclolignans as potential dual tubulin‐topoisomerase II inhibitors
Source: Arch Pharm (Weinheim). 2024 Nov 12;358(1):e2400600. doi: 10.1002/ardp.202400600 (PMC11726159; doi:10.1002/ardp.202400600)
Supplement: Supplementary file 1 — Supporting information. [file ARDP-358-e2400600-s002.doc]

**Supplemental Material: Novel Compounds and Biological Screening Results**

**Insights into podophyllotoxin lactone features: new cyclolignans as potential dual tubulin-topoisomerase II inhibitors.**

Ángela-Patricia Hernández^1,2*^, Celia Rosales-Fernández^1^, Carolina Miranda-Vera^1^, Anzhela Veselinova^3^, Pablo G. Jambrina^3^, Pilar García-García^1^, Pablo A. García^1^, David Díez^4^, Mª Ángeles Castro^1*^ and Manuel Fuentes^2,5^

1 Departamento de Ciencias Farmacéuticas, Área de Química Farmacéutica, Facultad de Farmacia, CIETUS/IBSAL, Universidad de Salamanca, Campus Miguel de Unamuno, 37007 Salamanca, Spain.

2 Department of Medicine and General Cytometry Service-Nucleus, CIBERONC CB16/12/00400, Cancer Research Centre (IBMCC/CSIC/USAL/IBSAL), IBSAL, Universidad de Salamanca-CSIC, Campus Miguel de Unamuno, s/n, 37007 Salamanca, Spain.

3 Departamento de Química Física, Facultad de Ciencias Químicas, Universidad de Salamanca, 37008 Salamanca, Spain.

4 Departamento de Química Orgánica, Facultad de Ciencias Químicas, Universidad de Salamanca, 37008 Salamanca, Spain.

5 Proteomics Unit, Cancer Research Centre (IBMCC/CSIC/USAL/IBSAL), 37007 Salamanca, Spain.

*Correspondence:

Ángela-Patricia Hernández and Mª Ángeles Castro, Departamento de Ciencias Farmacéuticas, Universidad de Salamanca, Campus Miguel de Unamuno, 37007 Salamanca, Spain.

Email: [angytahg@usal.es](mailto:angytahg@usal.es) (A.-P. H) and [macg@usal.es](mailto:macg@usal.es) (M. A. C.)

| **Compound No.** | **InChI** | **Biological Activity (IC_50_)** | |
| --- | --- | --- | --- |
|  |  | **Jurkat** | **HT-29** |
| **3** | InChI=1S/C31H29N3O7/c1-36-25-10-18(11-26(37-2)30(25)38-3)27-20-12-23-24(41-16-40-23)13-21(20)29(22-15-39-31(35)28(22)27)34-14-19(32-33-34)9-17-7-5-4-6-8-17/h4-8,10-14,22,27-29H,9,15-16H2,1-3H3/t22-,27+,28-,29+/m0/s1 | 8.59 ± 1.92 | 5.79 ± 0.17 |
| 4 | InChI=1S/C30H26FN3O7/c1-36-24-8-16(9-25(37-2)29(24)38-3)26-18-10-22-23(41-14-40-22)11-19(18)28(20-13-39-30(35)27(20)26)34-12-21(32-33-34)15-4-6-17(31)7-5-15/h4-12,20,26-28H,13-14H2,1-3H3/t20-,26+,27-,28+/m0/s1 | 8.97 ± 2.28 | 3.00 ± 0.26 |
| **5** | InChI=1S/C31H29N3O7/c1-36-25-10-18(11-26(37-2)30(25)38-3)27-20-12-23-24(41-16-40-23)13-21(20)29(22-15-39-31(35)28(22)27)34-14-19(32-33-34)9-17-7-5-4-6-8-17/h4-8,10-14,22,27-29H,9,15-16H2,1-3H3/t22-,27+,28+,29+/m0/s1 | 11.78 ± 1.62 | 4.85 ± 0.19 |
| **6** | InChI=1S/C30H26FN3O7/c1-36-24-8-16(9-25(37-2)29(24)38-3)26-18-10-22-23(41-14-40-22)11-19(18)28(20-13-39-30(35)27(20)26)34-12-21(32-33-34)15-4-6-17(31)7-5-15/h4-12,20,26-28H,13-14H2,1-3H3/t20-,26+,27+,28+/m0/s1 | 6.05 ± 2.73 | 1.25 ± 0.41 |
| **7** | InChI=1S/C31H30FN3O8/c1-38-25-9-17(10-26(39-2)30(25)40-3)27-19-11-23-24(43-15-42-23)12-20(19)29(21(14-36)28(27)31(37)41-4)35-13-22(33-34-35)16-5-7-18(32)8-6-16/h5-13,21,27-29,36H,14-15H2,1-4H3/t21-,27+,28+,29+/m0/s1 | 8.77 ± 0.27 | 0.94 ± 0.33 |
| **8** | InChI=1S/C33H32FN3O9/c1-17(38)44-15-23-30(33(39)43-5)29(19-10-27(40-2)32(42-4)28(11-19)41-3)21-12-25-26(46-16-45-25)13-22(21)31(23)37-14-24(35-36-37)18-6-8-20(34)9-7-18/h6-14,23,29-31H,15-16H2,1-5H3/t23-,29+,30+,31+/m0/s1 | 14.28 ± 1.74 | 1.36 ± 0.24 |
| **9** | InChI=1S/C31H28FN3O8/c1-38-25-9-17(10-26(39-2)30(25)40-3)27-19-11-23-24(43-15-42-23)12-20(19)29(21(14-36)28(27)31(37)41-4)35-13-22(33-34-35)16-5-7-18(32)8-6-16/h5-14,21,27-29H,15H2,1-4H3/t21-,27+,28-,29+/m0/s1 | 8.97 ± 1.09 | **0.04 ± 0.01** |
