# Supplementary material for: Insights into podophyllotoxin lactone features: New cyclolignans as potential dual tubulin‐topoisomerase II inhibitors
Source: Arch Pharm (Weinheim). 2024 Nov 12;358(1):e2400600. doi: 10.1002/ardp.202400600 (PMC11726159; doi:10.1002/ardp.202400600)
Supplement: Supplementary file 2 — Supporting information. [file ARDP-358-e2400600-s001.pdf]

Insights into podophyllotoxin lactone features: novel cyclolignans as potential dual tubulin-topoisomerase II inhibitors.

Ángela-Patricia Hernández[1,2]*, Celia Rosales-Fernández[1], Carolina Miranda-Vera[1], Anzhela Veselinova[3], Pablo G. Jambrina[3], Pilar García-García[1], Pablo A. García[1], David Díez[4], Mª Ángeles Castro[1]* and Manuel Fuentes[2,5]

[1] Departamento de Ciencias Farmacéuticas, Área de Química Farmacéutica, Facultad de Farmacia, CIETUS/IBSAL, University of Salamanca, Campus Miguel de Unamuno, 37007 Salamanca, Spain

[2] Department of Medicine and General Cytometry Service-Nucleus, CIBERONC CB16/12/00400, Cancer Research Centre (IBMCC/CSIC/USAL/IBSAL), IBSAL, Universidad de Salamanca -CSIC, Campus Miguel de Unamuno, s/n, 37007 Salamanca, Spain.

[3] Departamento de Química Física, Facultad de Ciencias Químicas, Universidad de Salamanca, 37008 Salamanca, Spain.

[4] Departamento de Química Orgánica, Facultad de Ciencias Químicas, Universidad de Salamanca, 37008 Salamanca, Spain.

[5] Proteomics Unit, Cancer Research Centre (IBMCC/CSIC/USAL/IBSAL), 37007 Salamanca, Spain.

* Correspondence: angytahg@usal.es (A.-P. H) and macg@usal.es (M. A. C.)

Supplementary Materials

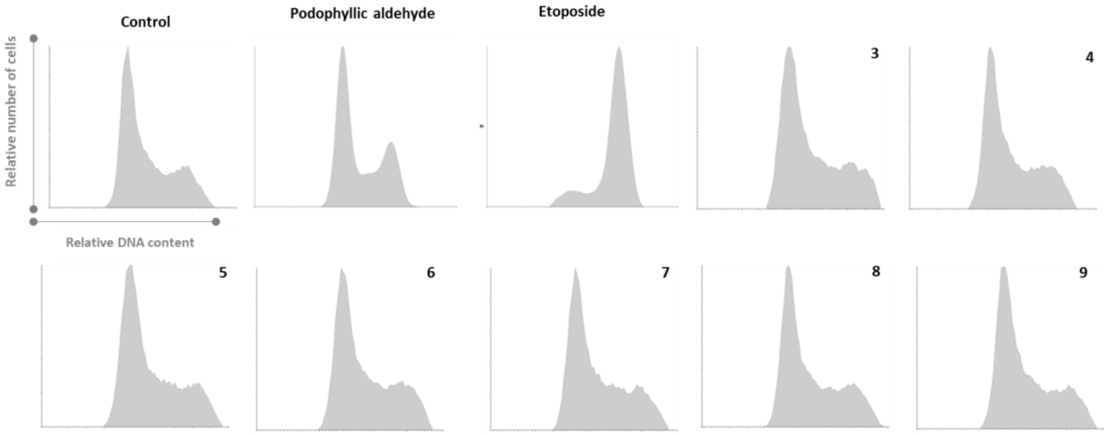

Figure S1: Cell cycle results for compounds **3-9** and positive controls (podophyllic aldehyde and etoposide) at 1 µM at 24 h Jurkat cells.

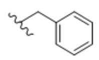

3, 5

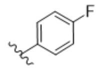

4, 6

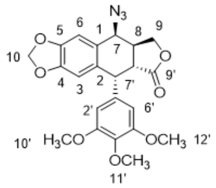

2

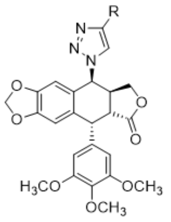

3, 4

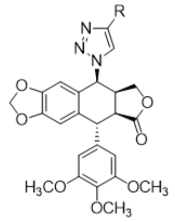

5, 6

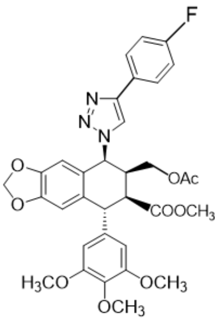

8

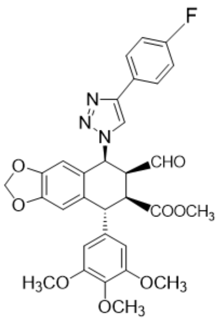

9

Table S1: ^{13}C NMR for compound 2-6, 78 and 9, δ in ppm.

^{13}C	**2**	**3**	**4**	**5**	**6**	**8**	**9**
1	132.1	134.3	133.3	134.1	130.2	132.0	131.5
2	126.8	133.1	131.2	130.4	127.7	126.4	126.5
3	111.1	110.4	110.4	110.3	110.3	110.2	110.2
4	147.3	147.7	147.0	147.5	147.9	147.4	147.6
5	149.0	143.0	148.0	148.4	148.6	148.7	148.6
6	108.7	105.3	106.6	106.3	106.6	106.1	106.5
7	59.5	67.4	69.3	69.4	69.2	62.6	69.3
8	36.7	41.3	38.3	38.5	38.3	37.6	38.3
9	67.6	60.7	60.0	60.9	60.9	60.9	199.1
10	101.8	101.9	101.7	101.6	101.7	101.6	101.7
1’	135.0	137.6	137.4	137.2	137.3	137.2	137.3
2’6’	108.3	108.2	104.8	104.6	104.8	107.6	107.0
3’5’	152.6	153.7	157.8	153.4	153.7	153.4	153.1
4’	137.4	138.5	137.4	137.6	138.2	139.4	138.1
7’	41.2	43.2	43.0	43.3	44.8	45.1	46.2
8’	43.7	47.9	45.3	45.4	45.3	47.5	49.5
9’	174.0	173.2	175.4	177.7	177.5	172.7	171.6
10’ 12’	56.27	58.2	56.4	56.2	56.3	56.3	56.3
11’	60.4	58.5	59.7	59.1	59.3	59.7	60.7
Triazole	-	121.6<br>126.6	120.1<br>128.7	122.5<br>126.7	120.4<br>126.1	120.1<br>126.4	120.2<br>126.5
R	-	32.3<br>124.9<br>128.6<br>128.7<br>128.8	115.9<br>116.1<br>127.7<br>127.6<br>161.7<br>164.2	32.1<br>126.6<br>128.2<br>128.3<br>128.7	115.8<br>116.1<br>127.6<br>127.7<br>161.6<br>164.1	115.8<br>116.0<br>127.3<br>127.4<br>161.5<br>163.9	115.8<br>116.0<br>127.4<br>127.5<br>161.5<br>164.0
9’-OCH3	-	-	-	-	-	52.1	52.6
9-OAc	-	-	-	-	-	20.7<br>170.4	-

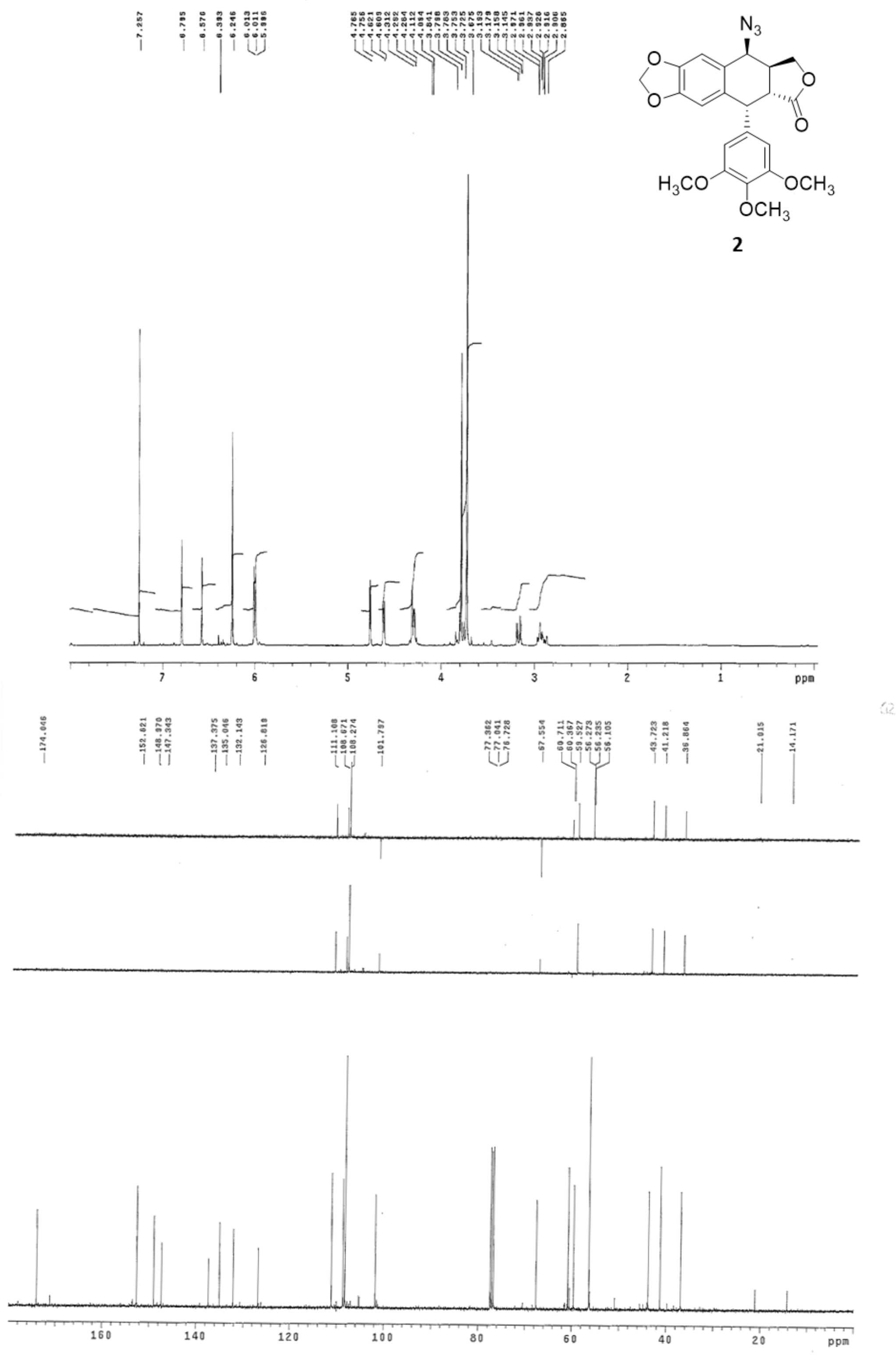

Figure S2: ^{1}H and ^{13}C NMR spectra for compound **2.**

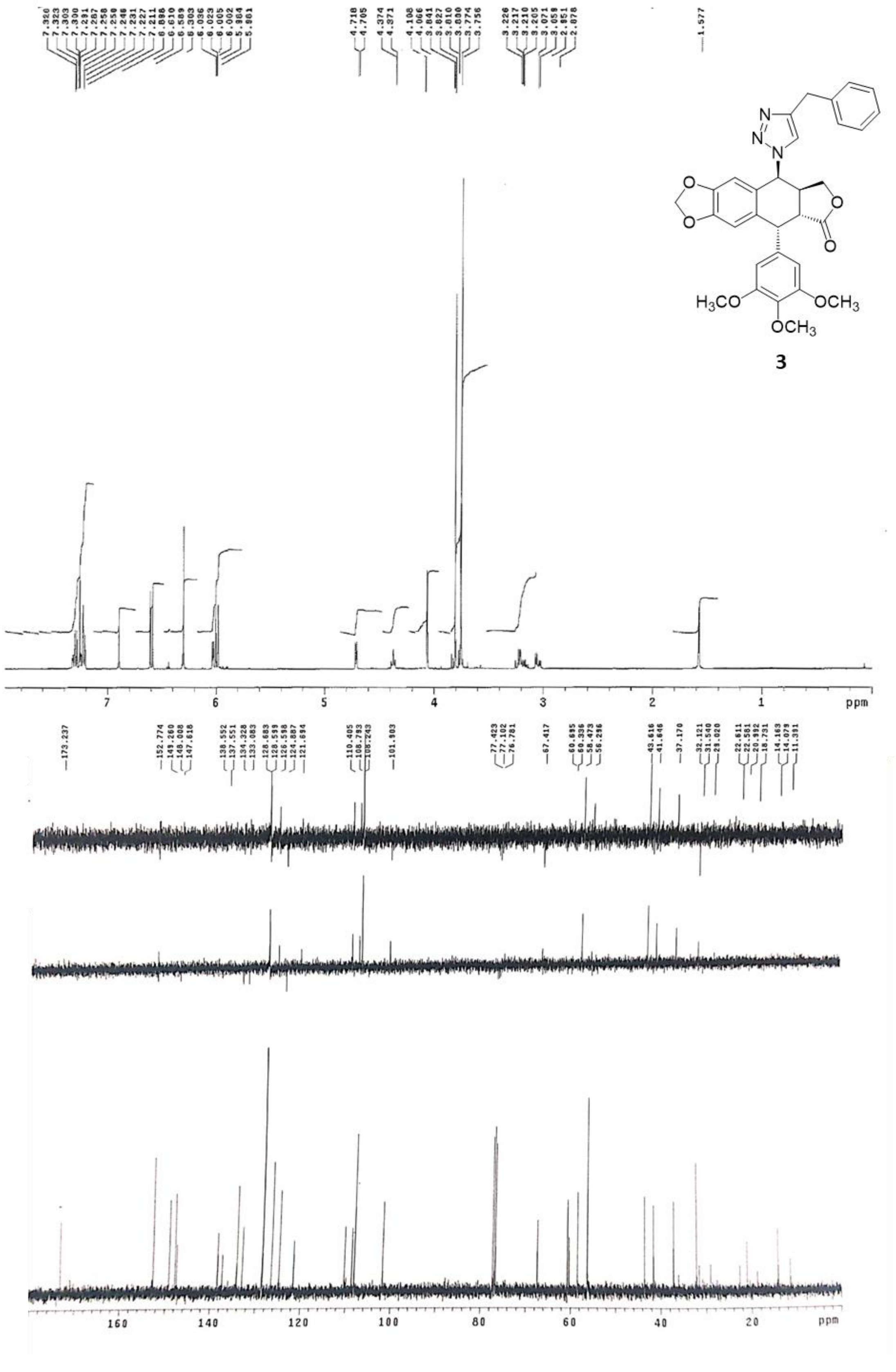

Figure S3: ^{1}H and ^{13}C NMR spectra for compound **3.**

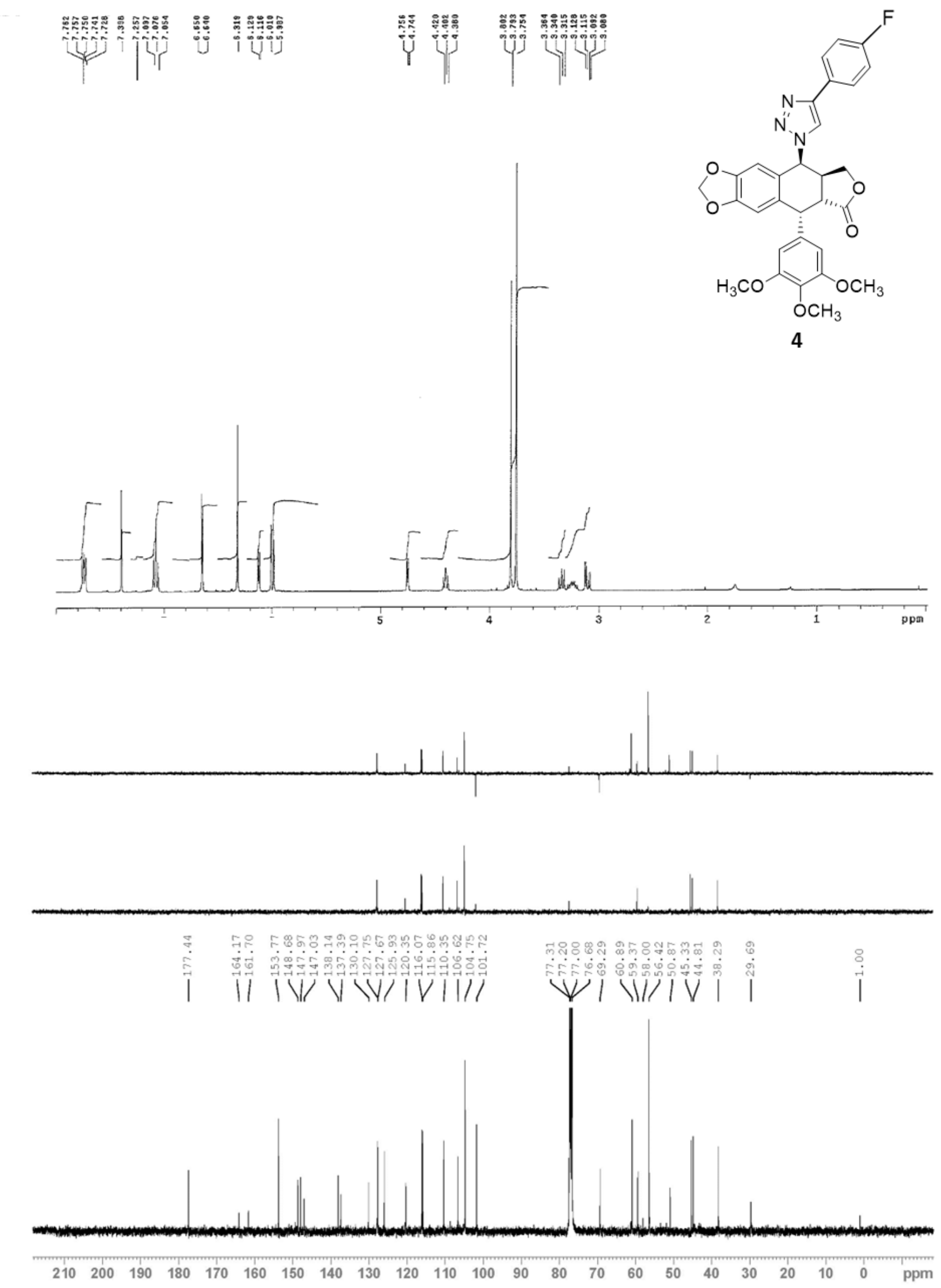

Figure S4: ^{1}H and ^{13}C NMR spectra for compound **4.**

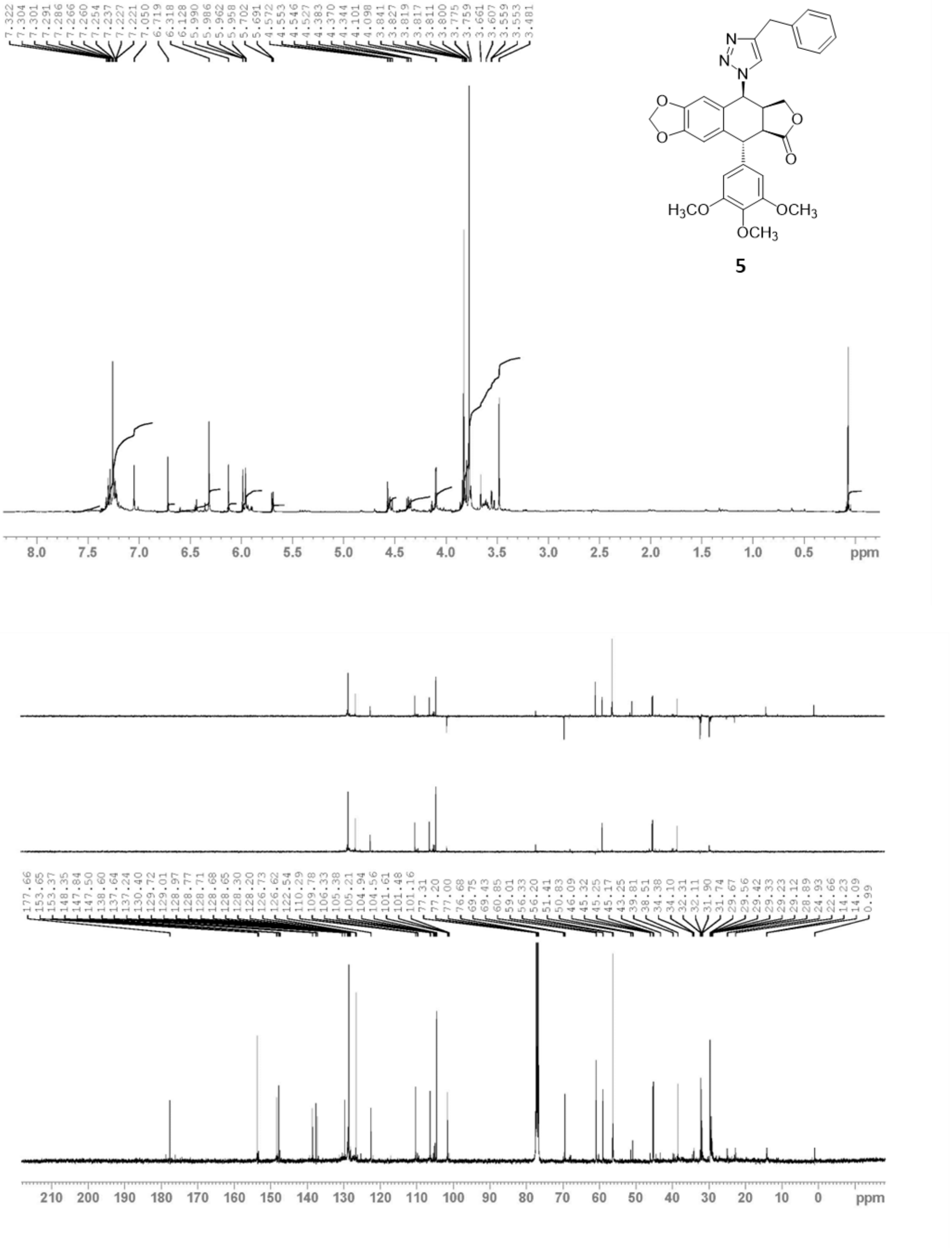

Figure S5: ^{1}H and ^{13}C NMR spectra for compound **5.**

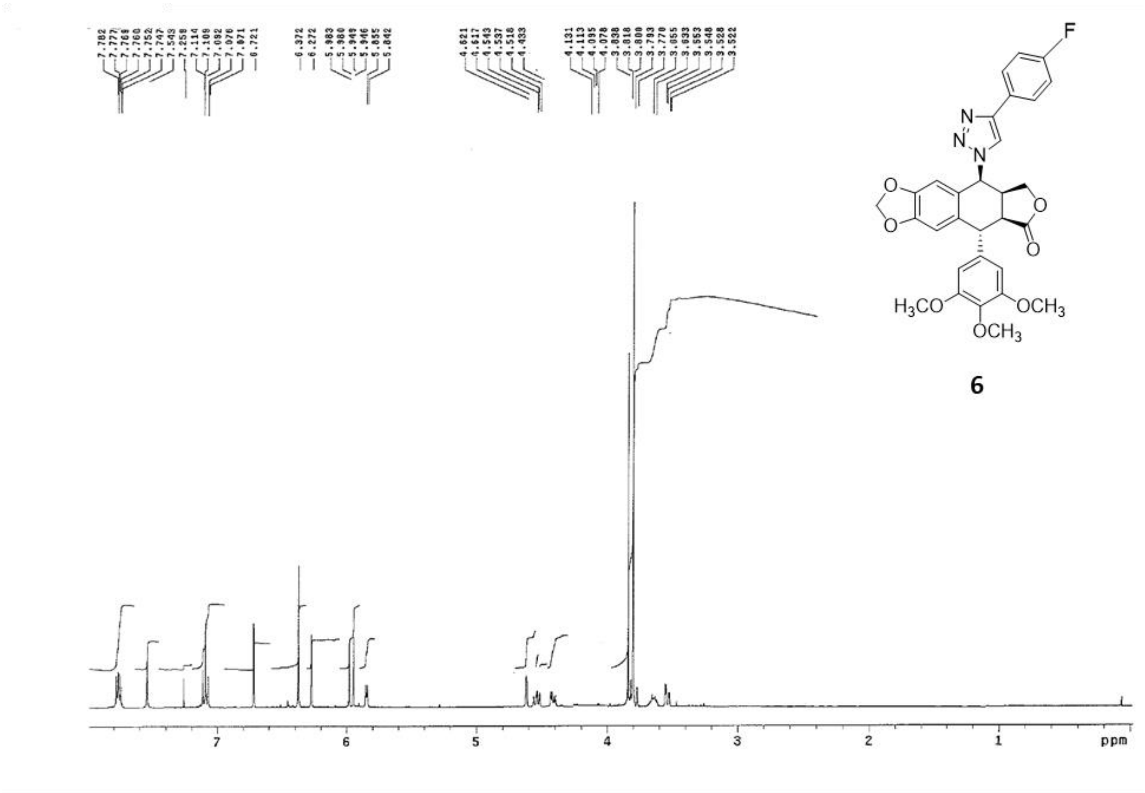

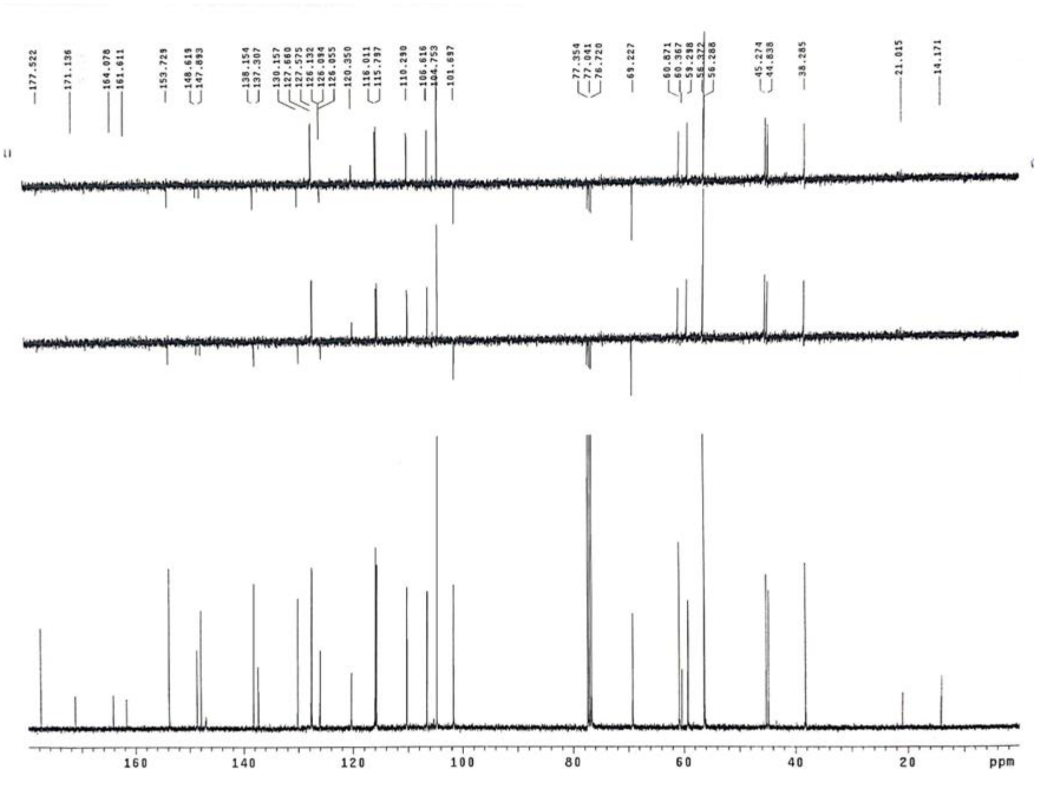

Figure S6: ^{1}H and ^{13}C NMR spectra for compound **6.**

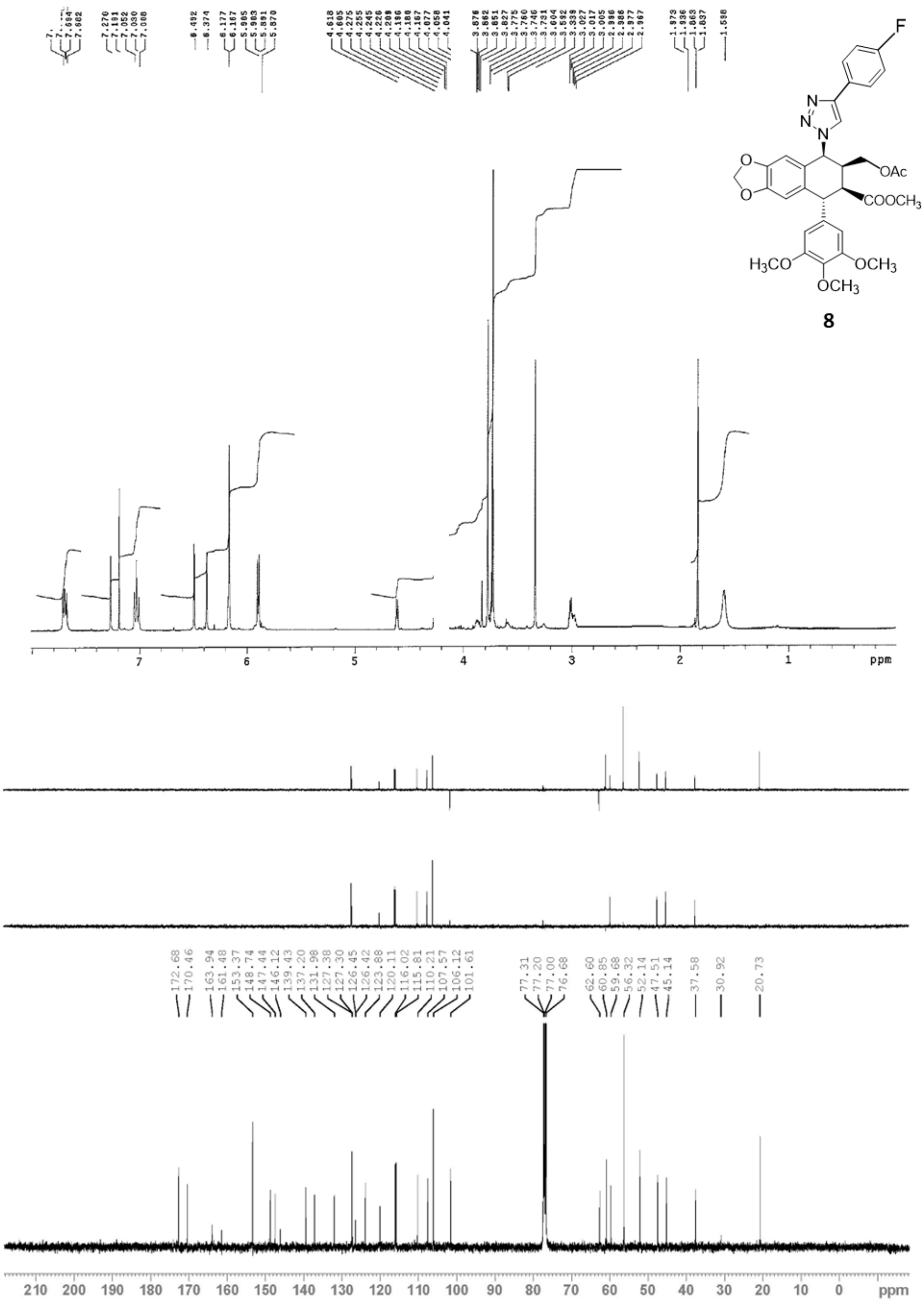

Figure S7: ^{1}H and ^{13}C NMR spectra for compound **8.**

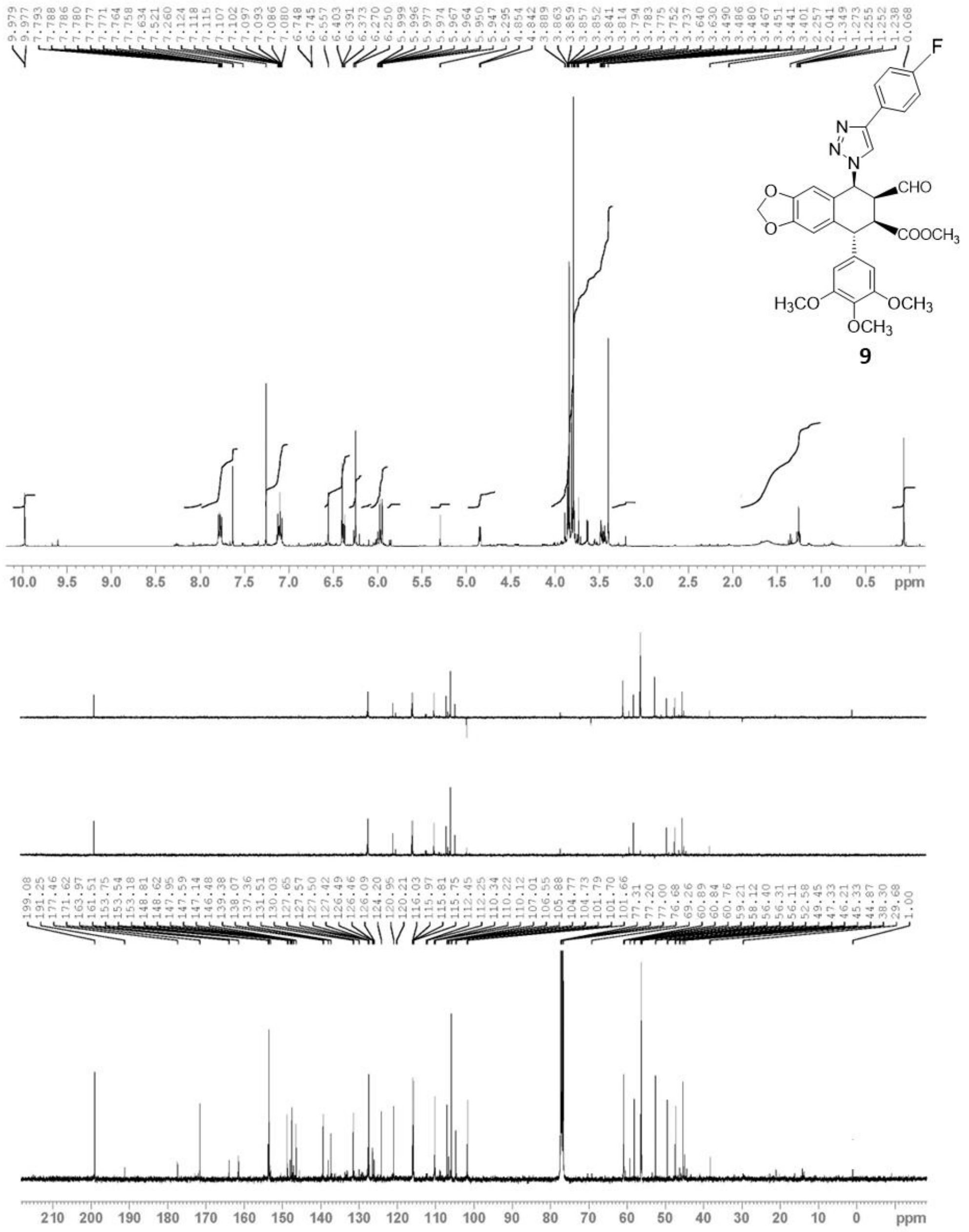

Figure S8: ^{1}H and ^{13}C NMR spectra for compound **9.**

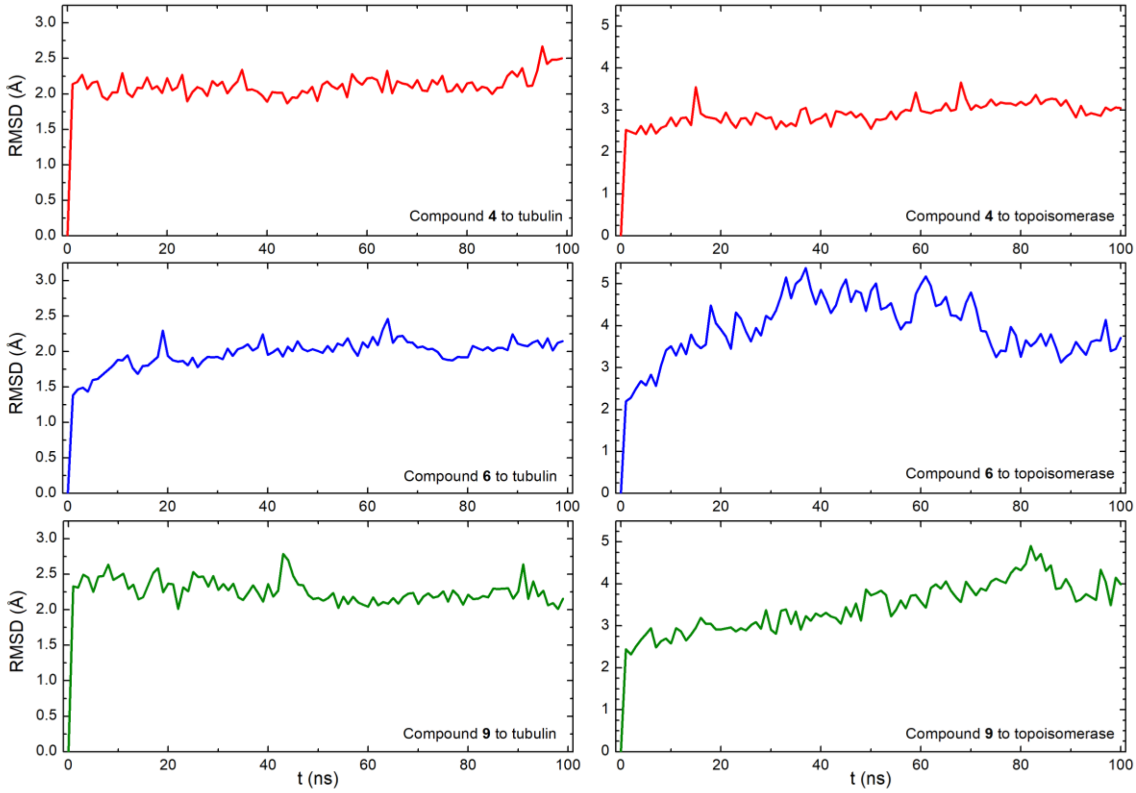

Figure S9: RMSD of the protein along the MD simulations
